# Proteins in Ionic Liquids: Reactions, Applications, and Futures

**DOI:** 10.3389/fchem.2019.00347

**Published:** 2019-05-24

**Authors:** Alexandra Schindl, Matthew L. Hagen, Shafaq Muzammal, Henadira A. D. Gunasekera, Anna K. Croft

**Affiliations:** ^1^Department of Chemical and Environmental Engineering, Faculty of Engineering, University of Nottingham, Nottingham, United Kingdom; ^2^Faculty of Medicine & Health Sciences, School of Life Sciences, University of Nottingham, Nottingham, United Kingdom; ^3^Faculty of Science, School of Pharmacy, University of Nottingham, Nottingham, United Kingdom; ^4^Centre for Additive Manufacturing, Faculty of Engineering, University of Nottingham, Nottingham, United Kingdom

**Keywords:** ionic liquids, enzymes, proteins, intermolecular interactions, reactions, molecular organization, physico-chemical relationships

## Abstract

Biopolymer processing and handling is greatly facilitated by the use of ionic liquids, given the increased solubility, and in some cases, structural stability imparted to these molecules. Focussing on proteins, we highlight here not just the key drivers behind protein-ionic liquid interactions that facilitate these functionalities, but address relevant current and potential applications of protein-ionic liquid interactions, including areas of future interest.

## Introduction

Proteins as both materials and catalysts have a number of practical features when considering global challenges such as developing a circular economy and minimization of environmental impacts. They are biodegradable and can be readily biosourced, are renewable, and can act as biocompatible scaffolds. Being polymeric materials consisting of combinations of around 20 main building blocks (amino acids), the range of materials properties that are accessible is substantial. The different amino acid functionalities lend these polymers to further post-processing, which can further extend the property scope. In addition to their catalytic properties as enzymes, proteins such as keratins, collagens, silks, and plant-fibers are strong, can be highly elastic, and possess many other desirable materials properties, including being suitable scaffolds for living cells. As such, these biopolymers have found significant use in the medical industries in particular, due to this biocompatibility and ability to replace or enhance existing tissues (Defrates et al., 2018).

Ionic liquids have firmly established themselves as useful industrial and laboratory solvents, reflected by substantial and ever-increasing literature in the area. Due to the reduced vapor pressure, arising from the strong electrostatic interactions of the constituent ions, they possess a number of useful properties, some of which underpin their “green” reputation. This includes minimal harmful vapor when handling, low flammability, and the lack of vaporization, which gives an opportunity to recycle these liquids across many cycles. The number of different ion combinations that can be considered leads to over 10^6^ potential ionic liquids (Rogers and Seddon, 2003), before mixtures are even considered, each with different physical and chemical properties. Thus, in principle, tuning of the properties for a particular task can be achieved through judicious selection of ion combinations. They are especially good for the dissolution of recalcitrant materials, as the combination of electrostatic, hydrogen-bonding, π- and van der Waals interactions means that non-covalent (and sometimes covalent) interactions within these materials are more readily broken, with concomitant stabilization in the solution form.

**Figure 1:**
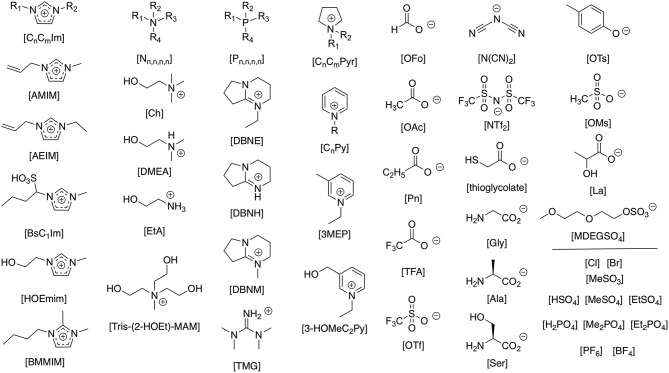
Representative ionic liquid cation and anion structures presented throughout the text.

The interaction of ionic liquids with proteins adds a significant new landscape for the understanding of ionic-liquid solute interactions. With the vast range of cation and anion combinations available (see Figure 1 for those highlighted in this review) affording a differing balance of intermolecular interactions and thus interacting properties that can constitute an ionic liquid, not to mention mixtures of ions, the different anionic, cationic, hydrophobic, and polar interactions from each amino acid of a protein backbone becomes a many dimensional challenge.

## Structural Proteins

### Keratins

Keratins are proteins that can be sourced from the skin, hair, horns, nails, beaks, and teeth of different mammals, reptiles, birds, and fish (Mckittrick et al., 2012), and constitute a significant biowaste stream (Zoccola et al., 2009; Sharma and Gupta, 2016; Holkar et al., 2018). As a natural fiber, wool is widely used commercially in the textile industry (Lewis and Rippon, 2013), and has found many uses ranging from biomedical and cosmetic applications (Rouse and Van Dyke, 2010; Silva et al., 2014; Sharma and Gupta, 2016), (being documented in medical use at least since ancient Greek times) (Alves et al., 2013), to fertilizers and flame retardants (Sharma and Gupta, 2016).

Keratins can be categorized as either hard or soft, depending on their sulfur content, with hard keratins, like wool, having a high sulfur content, and therefore more covalent (disulfide) crosslinks (Simpson and Crawshaw, 2002; Zoccola et al., 2009; Mckittrick et al., 2012). Soft keratins can be found in the outer layer of the skin and have lower sulfur content and concomitantly fewer covalent (disulfide) crosslinks (Zoccola et al., 2009; Mckittrick et al., 2012). The disulfide bridges and strong inter- and intra-protein hydrogen-bonding of keratin proteins provide a significant processing challenge (Liu et al., 2018), often addressed through using mixtures of different solvents, with each solvent playing a key role in breaking covalent and/or non-covalent bonds (Xie et al., 2005). Volatility, corrosiveness, environmental impact, difficulty of recycling, and lack of renewability have all been highlighted as issues with these solvent regimes, in addition to the requirements for multi-step processes and resultant keratin degradation leading to regenerated keratin with a lower value (Hameed and Guo, 2009; Idris et al., 2013; Ji et al., 2014; Zheng et al., 2015; Liu et al., 2017; Zhang et al., 2017b). As such, there has been a growing demand to develop new solvents for keratin dissolution that are able to overcome the disadvantages associated with the traditional/existing solvents.

Ionic liquids have naturally been examined as solvents for the post-processing of keratin (Xie et al., 2005; Hameed and Guo, 2009; Sun et al., 2009; Zhao et al., 2010b; Lovejoy et al., 2012; Wang and Cao, 2012; Idris et al., 2013, 2014a,b; Li and Wang, 2013; Chen et al., 2014, 2015; Ghosh et al., 2014; Ji et al., 2014; Plowman et al., 2014; Wang et al., 2014, 2016; Zheng et al., 2015; Kammiovirta et al., 2016; Sharma and Gupta, 2016; Tran and Mututuvari, 2016; Liu et al., 2017, 2018; Zhang et al., 2017a;Zhang et al., 2017b). The dissolution process of keratin fiber starts with the swelling of the cuticle (outer layer) followed by the swelling and preferential dissolution of the cortex (inner layer). Of the two components, the cuticle generally takes longer to dissolve due to the high number of disulfide bonds present (Chen et al., 2014), however, selective swelling of the cuticle retaining the cortex intact is possible through careful control of conditions (Chen et al., 2015). According to Wang et al. (2014) for imidazolium cation and chloride anion-based ionic liquids, the combined effect of the anion and cation is responsible for breaking the covalent and non-covalent interactions in keratin, with each component participating through different interactions with the substrate (Ghosh et al., 2014).

An extensive array of ionic liquids has been tested with keratins (Table 1), reflecting the interest in reprocessing this protein. Early on, [C_4_C_1_Im][Cl] gained significant traction as the ionic liquid of choice for keratin dissolution (Xie et al., 2005; Hameed and Guo, 2009; Sun et al., 2009; Lovejoy et al., 2012; Idris et al., 2013; Li and Wang, 2013; Chen et al., 2014; Ghosh et al., 2014; Ji et al., 2014; Plowman et al., 2014; Wang et al., 2014; Zheng et al., 2015; Tran and Mututuvari, 2016; Zhang et al., 2017a; Liu et al., 2018), being effective for a broad range of keratin types, including human hair, hooves, goat and sheep wool, and duck, turkey and chicken feathers. Other small-cation imidazolium ionic liquids, primarily [AMIM], [C_2_C_1_Im] and [C_4_C_1_Im]-based derivatives have also been extensively trialed. It is the role of the anion, however, that appears to be key in the quality of regenerated keratin, with the nucleophilicity of chloride being suggested as the reason behind the degradation particularly of the disulfide bonds, and subsequent formation of cysteic acid (Ghosh et al., 2014). Acetate has also been strongly implicated in this process and was more effective than chloride, both for keratin and model systems (Zhang et al., 2017a). The differences between different cations with the same anion may potentially be attributed to the differences in ion-ion interactions providing a different effective availability of the corresponding anion. The wider ionic liquid organizational structure, combined with the specific structuring of the keratin, may also have implications for selectivity of different disulfides in the degradation process (Karimi et al., 2016).

**Table 1:** Summary of keratin solubility in selected ionic liquids.

**Ionic liquid**	**Keratin type**	**Solubility**	**Dissolution conditions**	**References**
[AMIM][Cl]	Wool keratin	8 wt%	130°C, N_2_, 10 h	Xie et al., 2005
	Turkey feathers	50 wt%	130°C, N_2_, 10 h	Idris et al., 2013
	Wool keratin	21 wt%	130°C, 10.5 h	Li and Wang, 2013
	Merino Wool	200 mg/g	130°C, N_2_, 10 h	Idris et al., 2014b
	Duck feather	5 wt%	120°C, 60 min	Ji et al., 2014
	Human hair	19 wt%	130°C	Wang et al., 2014
	Goat wool keratin	9 wt%	120°C, 30 min	Zhang et al., 2017a
	Wool keratin	13 wt%	120°C, 24 h	Liu et al., 2018
[AMIM][N(CN)_2_]	Merino Wool	475 mg/g	130°C, N_2_, 10 h	Idris et al., 2014b
[BsC_1_Im][HSO_4_]	Duck feather	4 wt%	120°C, 60 min	Ji et al., 2014
[C_1_Im][OTf]	Duck feather	< 1 wt%	120°C, 60 min	Ji et al., 2014
[C_2_C_1_Im][OAc]	Wool keratin	38 wt%	120°C, 24 h	Liu et al., 2018
	Chicken Feathers	5 wt%	130°C, 2.5 h	Kammiovirta et al., 2016
	Goat wool keratin	9 wt%	120°C, 30 min	Zhang et al., 2017a
[C_2_C_1_Im][Cl]	Wool keratin	14 wt%	120°C, 24 h	Liu et al., 2018
	Goat wool keratin	9 wt%	120°C, 30 min	Zhang et al., 2017a
[C_2_C_1_Im][Et_2_PO_4_]	Goat wool	8 wt%	120°C, 1.5 h	Liu et al., 2017
	Goat wool keratin	9 wt%	80-140°C,^c^ 30–180 min	Zhang et al., 2017a
	Wool keratin	22 wt%	120°C	Liu et al., 2018
[C_2_C_1_Im][Me_2_PO_4_]	Wool keratin	8 wt%	130°C, 1.5 h	Zheng et al., 2015
	Wool keratin	9 wt%	130°C	Zhang et al., 2017b
[C_4_C_1_Im][OAc]	Goat wool keratin	9 wt%	120°C, 30 min	Zhang et al., 2017a
	Goat wool	8 wt%	120°C, 2.5 h	Liu et al., 2017
	Wool keratin	8 wt%	130°C, 10 min	Zheng et al., 2015
	Wool	single fibers	120°C, 3 min	Chen et al., 2014
	Merino Wool	Cuticle removal	75°C, 35 min	Chen et al., 2015
	Goat wool keratin	9 wt%	120°C, 30 min	Zhang et al., 2017a
[C_4_C_1_Im][BF_4_]^†^	Wool keratin	n/d	130°C, N_2_, 24 h	Xie et al., 2005
	Human hair	insoluble	130°C	Wang et al., 2014
[C_4_C_1_Im][Br]	Wool keratin	2 wt%	130°C, N_2_, 10 h	Xie et al., 2005
	Duck feather	4 wt%	120°C, 60 min	Ji et al., 2014
	Human hair	10 wt%	130°C	Wang et al., 2014
[C_4_C_1_Im][Cl]	Wool keratin	11 wt%	130°C, N_2_, 10 h	Xie et al., 2005
	Wool	5 wt%	100°C	Hameed and Guo, 2009
	Chicken feather	23 wt%	100°C, N_2_, 48 h	Sun et al., 2009
	Pig hoof powder	1 wt%	130°C, 10 h	Lovejoy et al., 2012
	Turkey feathers	50 wt%	130°C, N_2_, 10 h	Idris et al., 2013
	Wool keratin	15 wt%	130°C, 9 h	Li and Wang, 2013
	Duck feather	5 wt%	120°C, 60 min	Ji et al., 2014
	Human hair	13 wt%	130°C	Wang et al., 2014
	Wool	single fibers	120°C, 90 min	Chen et al., 2014
	Merino Wool	5 wt%	99°C, 18 h	Plowman et al., 2014
	Merino Wool fabric	14 wt%	120, 150, and 180°C, 30 min	Ghosh et al., 2014
	Merino Wool	250 mg/g	130°C, N_2_, 10 h	Idris et al., 2014b
	Wool keratin	8 wt%	130°C, 5 h	Zheng et al., 2015
	Raw wool	6 wt%	125–130°C, 6–8 h	Tran et al., 2016
	Goat wool keratin	9 wt%	120°C, 30 min	Zhang et al., 2017a
	Wool keratin	35 wt%	120°C, 24 h	Liu et al., 2018
[C_4_C_1_Im][Bu_2_PO_4_]	Wool	5.0% ^a^	120°C, N_2_, 12 h	Wang et al., 2016
[C_4_C_1_Im][N(CN)_2_]	Wool keratin	1.5 wt%	120°C, 24 h	Liu et al., 2018
[C_4_C_1_Im][Me_2_PO_4_]	Wool keratin	8 wt%	130°C, 1.5 h	Zheng et al., 2015
[C_4_C_1_Im][H_2_PO_4_]	Wool	5.0% ^a^	120°C, N_2_, 10.5 h	Wang et al., 2016
	Wool	5.0%^a^	120°C, N_2_, 17 h	Wang et al., 2016
[C_4_C_1_Im][HSO_4_]	Wool	5.0% ^a^	120°C, N_2_, 13.5 h	Wang et al., 2016
[C_4_C_1_Im][NO_3_]	Duck feather	4 wt%	120°C, 60 min	Ji et al., 2014
[C_4_C_1_Im][SCN]	Wool keratin	8 wt%	130°C, 15 h	Zheng et al., 2015
	Wool keratin	< 1 wt%	120°C, 24 h	Liu et al., 2018
[C_4_C_1_Pyr][Cl]	Wool keratin	40 wt%	120°C, 24 h	Liu et al., 2018
	Pig hoof powder	1 wt%	130°C, 10 h	Lovejoy et al., 2012
[C_4_Py][Cl]	Wool keratin	36 wt%	120°C, 24 h	Liu et al., 2018
[Ch][thioglycolate]	Turkey feathers	45 wt%	130°C, N_2_, 10 h	Idris et al., 2013
	Merino Wool	225 mg/g	130°C, N_2_, 10 h	Idris et al., 2014b
	Wool	single fibers	120°C, 10 min	Chen et al., 2014
[Ch][Pn]	Wool	single fibers	120°C, 45 min	Chen et al., 2014
	Merino Wool	Cuticle removal	75°C, 35 min	Chen et al., 2015
[DBNE][Et_2_PO_4_]	Goat wool	8 wt%	120°C, 3 h	Liu et al., 2017
[DBNH][OAc]	Goat wool	8 wt%	120°C, 20 min	Liu et al., 2017
[DBNM][Me_2_PO_4_]	Goat wool	8 wt%	120°C, 3.5 h	Liu et al., 2017
[DMEA][HCO_2_]	Turkey feathers	150 mg/g	100°C, 7 h	Idris et al., 2014a
[HOEmim][Cl]	Wool keratin	3 wt%	120°C, 24 h	Liu et al., 2018
[HOEmim][NTf_2_]	Chicken feathers	21.75%^b^	80°C, 4 h	Wang and Cao, 2012
[N_2, 2, 2, 1_][Me_2_PO_4_]	Wool keratin	8 wt%	130°C, 3 h	Zheng et al., 2015
[P_4, 4, 4, 4_][Cl]	Pig hoof powder	1 wt%	130°C, 10 h	Lovejoy et al., 2012
[TMG][Pn]	Wool	single fibers	100°C, 6.5 h, partial dissolution	Chen et al., 2014
	Merino Wool	Cuticle removal	75°C, 35 min	Chen et al., 2015

a *wool fiber/ionic liquid mass ratio*.

b *extraction yield based on 1:45 mass ratio*.

c *No dissolution reported below 110°C*.

*Bs, 1-sulfobutyl; Pn, propionate; OAc, acetate; DBNE, 1-Ethyl-1,5-diazabicyclo[4.3.0]-non-5-enium; DBNH, 1,5-diazabicyclo [4.3.0]non-5-enium; DBNM, 1-methyl-1,5-diazabicyclo[4.3.0]non-5-enium; DMEA, N,N-dimethylethanolammonium; TMG, 1,1,3,3-Tetramethylguanidinium*.

† *Hydrolysable ionic liquid*.

There has been a growing drive to expand the range of suitable solvents for keratin to those that have specific properties useful for larger-scale processing, and, as such, both distillable and protic ionic liquids have been utilized with good effect (Chen et al., 2014, 2015; Idris et al., 2014a), alongside those containing potentially more benign, bio-compatible (Lovejoy et al., 2012; Zheng et al., 2015) and bio-derived (Idris et al., 2013; Chen et al., 2014, 2015), cations. Ionic liquids that have been tested and were reported to show either very poor or no detectable solubility of keratins include: [C_4_C_1_Im][PF_6_] (Xie et al., 2005), [C_1_Im][Cl] (Zhang et al., 2017a), [C_4_Py][Cl] (Zheng et al., 2015; Zhang et al., 2017a), [C_4_C_1_Im][Br] (Zhang et al., 2017a), [C_4_C_1_Im][DBP] (Zhang et al., 2017a), [P_4, 4, 4, 4_][Cl] (Zheng et al., 2015; Zhang et al., 2017a), [N_4, 4, 4, 4_][Cl] (Zheng et al., 2015; Zhang et al., 2017a), [C_4_C_1_Im][H_2_PO_4_] (Zhang et al., 2017a), [C_4_C_1_Pyr][NTf_2_] (Lovejoy et al., 2012), [C_4_C_1_Im][OctSO_4_] (Lovejoy et al., 2012), [P_6, 6, 6, 14_][N(CN)_2_] (Lovejoy et al., 2012), [P_1, sec−4, sec−4, sec−4_][OTs] (Lovejoy et al., 2012), [C_8_C_1_Im][BF4] (Lovejoy et al., 2012), [P_6, 6, 6, 14_][Cl] (Lovejoy et al., 2012), [P_6, 6, 6, 14_][NTf_2_] (Lovejoy et al., 2012), [P_6, 6, 6, 14_][BF_4_] (Lovejoy et al., 2012), [C_1_Im][BF_4_] (Lovejoy et al., 2012), [C_4_C_1_Im][BF_4_] (Xie et al., 2005; Lovejoy et al., 2012) and [C_4_C_1_Im][FeCl_4_] (Zheng et al., 2015). The ionic liquids [DMEA][OAc] and [DMEA][Cl] were also identified as potential candidates for dissolution, but because they had poor processing parameters, have not been tested for solubility (Idris et al., 2014a).

Recently a more systematic approach to determining useful ionic liquids for keratin dissolution was successfully applied, exploiting computational prediction of ionic liquid properties (Keaveney et al., 2015), based on the parametrised COSMO-RS methodology, with subsequent experimental verification (Liu et al., 2018). Here the σ-potential, a measure indicating the hydrogen bond acceptor-donor interactions of the model substrate, was matched for each model to generate the logarithmic activity coefficient (ln γ, proportional to the ionicity of the solution; Marsh et al., 1955), calculated against ionic liquid ion pairs. By testing 621 ion pairs and ranking these by cation and anion, a strong reliance on the anion was identified with a much more subdued contribution from the cation. Cations with additional hydrogen bonding groups, such as hydroxyethyl-methylimidazolium, acted to improve solubility over that predicted, and highlighted the role of hydrogen bond disruption in solubilisation.

### Collagens

Unlike the disulfide-reinforced, arginine/glutamate-rich keratins, collagens are predominantly glycine-rich proteins, featuring also proline and/or hydroxyproline. Collagens contribute a range of structural roles in skin, ligaments, cartilage and tendons, as well as in bones, teeth and the cornea (Chen et al., 2017). Three strands of collagen-protein are able to form stable triple-helicies, with macrostructural alignment further imparting specific mechanical properties (Chen et al., 2017), attractive for biomaterials development (Defrates et al., 2018). Since collagen has different hierarchical, ordered layers to keratin, it provides an alternative framework for understanding the interactions of different ionic liquids on peptide dissolution and stabilization.

Choline salts have been examined in the context of collagen, because they provide potential for more biologically benign interaction agents suitable for biological implants. Cross-linking has been achieved in [Ch][lactate] and [Ch][levulinate] (Vijayaraghavan et al., 2010), along with the related, non-liquid, [Ch][tartrate] (Vijayaraghavan et al., 2010) and [Ch][H_2_PO_4_] (Vijayaraghavan et al., 2010; Mehta et al., 2015). The primary mechanism of the cross-linking is facilitated by the strong electrostatic interactions between the protein (Meng et al., 2012), postulated as from the arginine residues, and the ionic liquid, specifically the anion (Mehta et al., 2015). This was further explored both experimentally and theoretically with [Ch]_2_[SO_4_] (Tarannum et al., 2018a), and subsequently a selection of amino acid-based cholinium salts, [Ch][Ser], [Ch][Thr], [Ch][Lys], and [Ch]Phe] (Tarannum et al., 2018b). In all these cases, the cholinium-based ionic liquids showed some variation in their interaction with collagen, based on features such as increases in the thermal denaturation temperature, fibril morphology, and changes in FTIR spectra, with more destabilization of the structure indicated for the lysinate and phenylalanate anions.

This dominance of the anionic interaction in governing stabilization is, however, challenged by the observation that [P_4, 4, 4, 1_][Et_2_PO_4_], [P_4, 4, 4, 1_][MeSO_3_], and [HN_2, 2, 1_][MeSO_3_] can result in structural deformation (Tarannum et al., 2018b), with [C_4_C_1_Im][Me_2_PO_4_] resulting in collagen structural degradation (Tarannum et al., 2018a), rather than strengthening of the interactions. The implication is that both the nature of the anion is strongly important, and that the cation must also play a not insignificant role. Physicochemical impacts, including on thermal denaturation, were also observed for aqueous solutions of imidazolium chlorides [C_2_C_1_Im][Cl], [C_4_C_1_Im][Cl], and [C_10_C_1_Im][Cl] (Mehta et al., 2014).

Collagen fiber solution has been utilized extensively in a broader range of imidazolium ionic liquids. In addition to [C_4_C_1_Im][Cl] (Meng et al., 2012; Wang et al., 2013a; Mehta et al., 2014), where around 6 wt% collagen could be solubilised, [C_2_C_1_Im][OAc] (Hu et al., 2013; Zhang et al., 2014) showed temperature-dependant solubilisation ranging from 3.1 to 7.4 wt% going from 25 to 45°C, respectively, which was also impacted, and sometimes improved (up to *ca*. 10 wt%) by addition of sodium salts (Hu et al., 2013). This ionic liquid has been utilized in extracting collagen from waste fish scales (Muhammad et al., 2017). Aqueous mixtures of [C_2_C_1_Im][EtSO_4_] have been used with gelatin (partially hydrolysed collagen) to prepare nanoparticle-embedded ionogels with a variety of attractive properties. Other biomedical applications have included composite hydrogels for skin dressings (Iqbal et al., 2017), and composite hydrogels for bone applications (Iqbal et al., 2018a), prepared with collagen using the protic [C_1_Im][OAc] as a carrier. The protic, non-imidazolium [NH(CH_2_CH_2_OH)_3_][OAc] has similarly been used for preparation of bone filler composites (Iqbal et al., 2018b).

Similar to the work in keratin, COSMO has been utilized in the calculation of activity coefficients with ionic liquids and a collagen model (Muhammad et al., 2017). Here a range of imidazolium cations; [C_2_C_1_Im], [C_4_C_1_Im], [C_6_C_1_Im], [C_8_C_1_Im], [C_10_C_1_Im]; with a large selection of corresponding anions was trialed, with lowest ln γ values obtained consistently for the smallest cation, especially in combination with small organic acid anions (acetate, propionate, formate, butanoate and crotonate, respectively). This was followed by the chloride anion, which interestingly had the largest variation in effect with cation, with a dramatic difference in ln γ values on going from [C_2_C_1_Im] to [C_4_C_1_Im] and again to [C_6_C_1_Im], compared with other cation-anion combinations. Amino acid anions [Gly]^−^, [Arg]^−^, and [Glu]^−^ afforded intermediate predictions, with more hydrophobic anions giving poor predictions for interaction, with high ln γ values. As a result, [C_2_C_1_Im][OAc] was selected in this study as extraction solvent of choice.

### Plant Proteins

The use of ionic liquids with plant proteins is less extensive than for those proteins from animals. The huge scale of soybean production worldwide means that soy protein is readily accessible (Nishinari et al., 2014). In practice soy protein is a mixture of different proteins of different sizes and properties, with two dominant, multimeric constituents, β-conglycinin and glycinin. These proteins are particularly rich in glutamate and aspartate, as well as arginine, lysine, serine, and proline, with higher quantities of leucine and phenylalanine in more hydrophobic subunits (Riblett et al., 2001). Soy protein has been used in a wet spinning method through dissolution in [C_4_C_1_Im][Cl]/DMSO mixtures (Deng et al., 2014), and blend films of soy protein with cellulose have been prepared with [AMIM][Cl] as the solvent (Wu et al., 2009).

The poorly-soluble zein protein is obtained as a by-product of industrial corn processing, most recently through bioethanol production (Gupta et al., 2016), and comprises around 7–8 wt% of the corn kernel (Shukla and Cheryan, 2001). Although rich in glutamate, leucine, alanine, and proline, it is low in basic amino acids such as arginine and deficient in lysine and tryptophan, making it poor quality for human nutrition. In an attempt to improve accessibility to reaction, nearly 15 wt% of zein was shown to be soluble in [C_4_C_1_Im][Cl], with practical viscosities in the range of 10 wt% when dissolved at 120°C for 60 min, and benzoylation able to be demonstrated (Biswas et al., 2006). The ionic liquid [C_4_C_1_Im][N(CN)_2_] was reported to be similarly effective with solubilities of 10 wt%, whereas [Ch][Cl] deep eutectic mixtures were not effective at dissolving zein. With a focus on assessing green solvents, the imidazolium ionic liquids [C_2_C_1_Im][OAc] and [C_2_C_1_Im][Br] were compared with bioderived organic solvents. Here 1,4-dioxane and 2,3-butanediol were indicated as more promising for zein extraction applications (Gupta et al., 2016), although other ionic liquids may have performed better had they been assessed.

In 2014, Tomlinson et al. (2014) examined zein solubility in seven imidazolium ionic liquids; four non protic: ([C_2_C_1_Im][OAc], [C_2_C_1_Im][N(CN)_2_], [C_4_C_1_Im][OAc], and [C_4_C_1_Im][Cl]) and three protic: ([C_1_Im][OAc], [C_1_Im][OFo], and [C_1_Im][HSO_4_]), and related their results to the Linear Free Energy parameters α, β, and π^*^ through multivariate regression. This model gave polarisability (π^*^) as the key contributor, increasing in importance at higher temperatures alongside the E_T_(30) polarity scale. They concluded that good solvents for zein would possess low hydrogen-bond accepting ability (β), low polarisability (π^*^) and weak hydrogen-bond donating ability (α), and that increases in water content within the ionic liquids had little impact on solubility. In line with this, they concluded that [C_1_Im][HSO_4_] was an extremely poor solvent and that [C_1_Im][OAc] and [C_2_C_1_Im][N(CN)_2_] were their preferred solvents for zein, with solubilities measured at *ca*. 25 wt% zein at 60°C. Other protic ionic liquids were also shown to be successful in solubilising zein with [NH_3_(CH_2_CH_2_OH)][OFo] and [NH_3_(CH_2_CH_2_OH)][OAc] producing highly viscous 70 wt% solutions (Choi and Kwon, 2011). More practical solutions of 20 wt% zein could be produced either by microwave heating, or by conventional heating at 120°C.

### Silks

Silk from the larval form of the silk moth *Bombyx mori* is a material that has been used by humanity for thousands of years. Whilst predominantly used for its properties as a textile, it has desirable mechanical properties (Pérez-Rigueiro et al., 2000), as well as potential biomedical applications (Reviewed Altman et al., 2003; Kapoor and Kundu, 2016), and is attracting significant research therefore in identifying mechanisms for reprocessing. Similar to the complex, layered structures of keratins, silk fibers are formed of two monofilaments called brins that are spun by the spinneret of the silk moth larve into a single fiber (bave). Each brin consists of two different proteinaceous substances: the core consists of fibroins and these are coated by serecins. For the sake of clarity when discussing solubility in ionic liquids, “silk” here will refer to fibroin obtained from *B. mori* with the serecin removed, unless explicitly stated.

The superlative mechanical characteristics of silk derive from a glycine- and alanine-rich repeating motif of the hexapeptide GAGAGX (where X = S, Y, A), periodically broken up by a GAAS motif (Zhou et al., 2001). Within silk fibers, these repeat regions make up a β-sheet structure interspersed with less structured regions. Further examinations of silk structure with small angle electron diffraction have shown that the β-sheets are organized into crystallites with dimensions of 66 ± 34 nm and 10 ± 3 nm with the major axis being aligned with the fiber axis (Shen et al., 1998), and it is this macrostructuring from the secondary level upwards that provides the underpinning mechanical properties of silk.

Previous attempts at solubilisation have required harsh solvents such as either hexafluoroisopropanol (HFIP) (Park et al., 2006), or aqueous inorganic salts, most commonly lithium bromide (Iizuka and Yang, 1966), that require subsequent dialysis to remove them afterward. Other methods have been explored for the dissolution of silk including calcium chloride in formic acid as well as mixtures of inorganic salts, water and alcohols (Yue et al., 2014; Cheng et al., 2015). In 2004, Phillips et al. (2004) published the first communication on the use of ionic liquids for silk dissolution, demonstrating [C_4_C_1_Im] and [C_2_C_1_Im]-based ionic liquids as effective solvents. Ionic liquids have since proven great utility in dissolving *B. mori* silk for a range of applications (Phillips et al., 2004, 2005; Gupta et al., 2007; Mantz et al., 2007; Goujon et al., 2012, 2013; Silva et al., 2012, 2013; Wang et al., 2012, 2013b; Zhou et al., 2013; Yao et al., 2014a,b, 2018; Li et al., 2015; Lozano-Pérez et al., 2015; Zhang et al., 2016a; Susanin et al., 2017, 2018; Stanton et al., 2018; Table 2). Successful dissolution has been achieved with a range of 1-methyl-3-alkylimidazolium ionic liquids, particularly when partnered with chloride or carboxylate anions. In a similar fashion to other proteins, maximal solubility is achieved with the small methyl and ethyl alkyl groups on these cations. This trend for smaller side chains does not extend to the protic [C_1_Im][Cl], in which silk is insoluble. Alkylation at the 2-position of the imidazole ring also decreases silk solubility. As this is a known site of H-bonding within the imidazolium ionic liquids, it implies that cation H-bonding is also a critical interaction in stabilizing silk solutions.

**Table 2:** Ionic liquid data for silk solubility found in the literature.

**IL**	**[Silk]/wt%**	**References**
[AMIM][Cl]	1–15	Wang et al., 2012
[C_1_Im][Cl]	Insoluble	Mantz et al., 2007
[C_2_Im][Cl]	Insoluble	Lozano-Pérez et al., 2015
[C_1_C_1_Im][Cl]	>12	Lozano-Pérez et al., 2015
[C_1_C_1_Im][NO_3_]	Insoluble	Mantz et al., 2007
[C_2_C_1_Im][Cl]	25	Lozano-Pérez et al., 2015
	23.3	Mantz et al., 2007
[C_2_C_1_Im][NO_3_]	Insoluble	Mantz et al., 2007
[C_2_C_1_Im][OAc]	0.1–20	Zhang et al., 2016a
[C_2_C_1_Im][SCN]	Insoluble	Mantz et al., 2007
[C_2_C_1_Im][OTf]	Insoluble	Mantz et al., 2007
		Lozano-Pérez et al., 2015
[C_2_C_1_Im][EtSO_4_]	Insoluble	Lozano-Pérez et al., 2015
[C_2_C_1_Im][BF_4_]	Insoluble	Mantz et al., 2007
[C_2_C_1_Im][AlCl_4_]	Insoluble	Mantz et al., 2007
[C_2_C_1_Im][Gly]	26.3	Mantz et al., 2007
[C_2_C_1_Im][Ala]	>20	Mantz et al., 2007
[C_2_C_1_Im][Ser]	>20	Mantz et al., 2007
[C_3_C_1_Im][Cl]	>15	Lozano-Pérez et al., 2015
[C_4_C_1_Im][Cl]	>12	Lozano-Pérez et al., 2015
	13.2	Phillips et al., 2004; Mantz et al., 2007
[C_4_C_1_Im][Br]	0.7	Phillips et al., 2004; Mantz et al., 2007^a^
[C_4_C_1_Im][I]	0.2	Phillips et al., 2004; Mantz et al., 2007^a^
[C_4_C_1_Im][OAc]	15	Li et al., 2015; Susanin et al., 2018
[C_4_C_1_Im][BF_4_]^†^	Insoluble	Phillips et al., 2004; Mantz et al., 2007^a^
[C_4_C_1_Im][PF_6_]^†^	Insoluble	Lozano-Pérez et al., 2015
[C_4_C_1_Im][OctSO_4_]	Insoluble	Lozano-Pérez et al., 2015
[C_4_C_1_Im][Cl]	8.3	Phillips et al., 2004; Mantz et al., 2007
[C_6_C_1_Im][Cl]	>11	Lozano-Pérez et al., 2015
[C_8_C_1_Im][Cl]	Insoluble	Lozano-Pérez et al., 2015
[C_10_C_1_Im][Cl]	Insoluble	Lozano-Pérez et al., 2015
[3-MEP][EtSO_4_]	Insoluble	Lozano-Pérez et al., 2015
EtAN	Insoluble	Lozano-Pérez et al., 2015
[N_4, 4, 4, 4_][Gly]	Insoluble	Mantz et al., 2007

a *Solubility data obtained from whole cocoons including serecin*.

*3-MEP, 3-methyl ethylpyridinium; EtAN, Ethanolammonium nitrate*.

† *Hydrolysable ionic liquid*.

From the surveyed ionic liquids, only three classes of anion have shown good solubilising properties: chloride, acetate, and amino acid anions. Although halides have shown to be effective anions, a direct comparison regarding solubility in [Cl]-based ionic liquids is difficult, as the [C_4_C_1_Im][X] (X = Br^−^, I^−^) ionic liquids were tested on whole cocoons including the serecin. These ionic liquids, as well as the hydrolysable [C_4_C_1_Im][BF_4_] (Freire et al., 2010), are capable of dissolving serecin whilst displaying minimal to no fibroin solubility, a trend also confirmed in later work (Mantz et al., 2007). The imidazolium acetates have been utilized extensively in cellulose dissolution (Swatloski et al., 2002), but interestingly it was not until 2016 before [C_2_C_1_Im][OAc] was used on silk (Zhang et al., 2016a). Particular anions (${\text{NO}}_{3}^{-}$, SCN^−^, TfO^−^, ${\text{EtSO}}_{4}^{-}$, ${\text{OctSO}}_{4}^{-}$, ${\text{BF}}_{4}^{-}$, ${\text{AlCl}}_{4}^{-}$, ${\text{PF}}_{6}^{-}$) do not appear to sufficiently perturb the H-bonding of silk to act as useful solvents.

Amino acid-based ionic liquids, where the [C_2_C_1_Im] cation was paired with the carboxylate anion form of the 20 natural amino acids, were initially developed by Fukumoto et al. (2005) with a subset of these ionic liquids characterized for their silk dissolution capacity (Table 2; Mantz et al., 2007). These amino acid anion-based ionic liquids provide some of the highest dissolution capacities for silk, showing promise in the area for more biocompatible dissolution solvents. One consideration however is whether the marginal gains in dissolution capacity, coupled to enhanced biocompatibility, are sufficient to justify the added complexity of their synthesis.

Solubility characterization has a direct impact on mechanisms of processing to afford silk in a desired functional form, such as the non-exhaustive examples highlighted in Table 3, which include composite formation for biomedical use (Silva et al., 2012). Silk coagulation from solution, although unsuccessful, also provides information on the underlying multi-way interactions between solvent, silk and coagulant. In general, small alcohols have been the most popular choice of coagulant, due to their ability to reform the β-sheet network critical to the strength of the silk II polymorph (Asakura et al., 1985). Water too has been utilized with differing levels of success where a regenerated silk will either not form at all ([C_4_C_1_Im][Cl] and 9.51 wt% silk, consistent with amorphous silk being soluble in water) or form brittle films, unless both cellulose is used as an additive and the correct ionic liquid ([C_2_C_1_Im][OAc] or [AMIm][Cl]) is chosen. Typical antisolvent choices for polar systems seem to yield either no coagulation or a brittle film or fiber (Table 3; Phillips et al., 2005). These observations emphasize that both solvent choice and the choice of coagulant are of paramount importance, with dominant options well-explored in the literature.

**Table 3:** A representative sample of methods by which silk is dissolved and subsequently reformed listing silk concentrations, additives, coagulants and processing methods.

**IL**	**[Silk] (wt%)**	**Additives**	**Coagulant**	**Processing method**	**RSF morphology**	**References**
[C_2_C_1_Im][Cl]	10.4	7% water	MeOH	Pipetted into coagulant bath, soaked for 24 h.	Fibers	Mantz et al., 2007
	10.4	7% water	EtOH	Pipetted into coagulant bath, soaked for 24 h.	Precipitate	Mantz et al., 2007
	10.4	7% water	0.1 M H_3_Ct−0.1 M NaH_2_Ct pH 2.96	Pipetted into coagulant bath, soaked for 24 h.	-	Mantz et al., 2007
	10.4	7% water	0.1 M NaH_2_Ct−0.1 M Na_2_HCt pH 4.29	Pipetted into coagulant bath, soaked for 24 h.	Precipitate	Mantz et al., 2007
	10.4	7% water	0.14 NaH_2_Ct−0.06M Na_2_HCt pH 4.05	Pipetted into coagulant bath, soaked for 24 h.	Precipitate	Mantz et al., 2007
	10	-	MeOH	Wet spinning.	Solid, clear fibers	Phillips et al., 2005
	10	-	MeCN	Wet spinning.	Solid, white crusted, brittle fibers	Phillips et al., 2005
	10	-	Water	Wet spinning.	Dissolved leaving small residual fiber	Phillips et al., 2005
	10	-	Acetone	Wet spinning.	Formed immiscible droplets, no precipitation	Phillips et al., 2005
	10	-	Ethyl Acetate	Wet spinning.	Formed immiscible droplets, no precipitation	Phillips et al., 2005
	10	-	Hexanes	Wet spinning.	Formed immiscible droplets, no precipitation	Phillips et al., 2005
	1	Cellulose 9 wt%	Water	Cast film between glass slides in coagulant bath.	Clear, solid film	Stanton et al., 2018
[C_2_C_1_Im][OAc]	0.1–20	-	Water	Gellation.	Gel	Zhang et al., 2016a
	5–10	-	Water	Gellation.	Conductive Gel	Yao et al., 2018
	5–10	-	Water/EtOH	Gellation.	Conductive Gel	Yao et al., 2018
	1	Cellulose 9 wt%	Water	Cast film between glass slides in coagulant bath.	Clear, solid film	Stanton et al., 2018
[AMIM][Cl]	1	Cellulose 9 wt%	Water	Cast film between glass slides in coagulant bath.	Clear, solid film	Stanton et al., 2018
[C_4_C_1_Im][Cl]	9.51	-	MeOH	Cast film in coagulant bath.	Transparent film, high crystallinity	Phillips et al., 2004, Mantz et al., 2007
	9.51	-	MeCN	Cast film in coagulant bath.	White film due to surface light scattering, low crystallinity	Phillips et al., 2004, Mantz et al., 2007
	9.51	-	Water	Cast film in coagulant bath.	Dissolved	Phillips et al., 2004, Mantz et al., 2007
	5	-	MeOH	Electrospun into coagulant bath with subsequent rinsing.	Fibers	Mantz et al., 2007
	1	Cellulose 9 wt%	Water	Cast film between glass slides in coagulant bath.	Clear, solid film	Stanton et al., 2018
	10	Water 25 wt%	MeOH	Spin Coating and immersion in coagulant.	Clear film	Gupta et al., 2007
	10	-	MeOH Vapor	Cast film in vacuum oven with MeOH vapor, then water rinse and pressed between glass plates and dried under reduced pressure.	Clear film	Zhou et al., 2013
	7.5	Cellulose 2.5 wt%	MeOH Vapor	Cast film in vacuum oven with MeOH vapor, then water rinse and pressed between glass plates and dried under reduced pressure.	Clear film	Zhou et al., 2013
	5	Cellulose 5 wt%	MeOH Vapor	Cast film in vacuum oven with MeOH vapor, then water rinse and pressed between glass plates and dried under reduced pressure.	Clear film	Zhou et al., 2013
	2.5	Cellulose 7.5 wt%	MeOH Vapor	Cast film in vacuum oven with MeOH vapor, then water rinse and pressed between glass plates and dried under reduced pressure.	Clear film	Zhou et al., 2013
	0	Cellulose 10 wt%	MeOH Vapor	Cast film in vacuum oven with MeOH vapor, then water rinse and pressed between glass plates and dried under reduced pressure.	Clear film	Zhou et al., 2013
[C_4_C_1_Im][Br]	1	Cellulose 9 wt%	Water	Cast film between glass slides in coagulant bath.	Translucent, brittle film	Stanton et al., 2018
[C_4_C_1_Im][OAc]	5	-	80% TMG.La: 20% water	Injected silk solution into coagulant bath, left for 1 hr and then rinsed 3x with water.	Silk foam	Goujon et al., 2012
	10	-	EtOH	Molds immersed in EtOH for 24 h then Soxhlet extraction with EtOH for 5 days then into MeOH/water (80/20 vol%) to form beta sheets.	Cast hydrogel	Silva et al., 2013
	2.8	Chitosan 1.2 wt%	EtOH	Molds immersed in EtOH for 24 h then Soxhlet extraction with EtOH for 3 days then into MeOH for 10 min to form beta sheets.	Cast hydrogel	Silva et al., 2012
	2	Chitosan 2 wt%	EtOH	Molds immersed in EtOH for 24 h then Soxhlet extraction with EtOH for 3 days.	Cast hydrogel	Silva et al., 2012
	1.2	Chitosan 2.8 wt%	EtOH	Molds immersed in EtOH for 24 h then Soxhlet extraction with EtOH for 3 days.	Cast hydrogel	Silva et al., 2012
	15	-	EtOH	1–2 h at 25°C 65% RH then into EtOH and finally into water.	Clear film	Li et al., 2015
[C_4_C_1_Im][MeSO_3_]	1	Cellulose 9 wt%	Water	Cast film between glass slides in coagulant bath.	Translucent, brittle film	Stanton et al., 2018

In contrast to water or alcohols as the coagulant for the regeneration of silk from solution, the tunability of ionic liquids offers an alternative approach to regeneration, and highlights the potential of these solvents. Protic ionic liquids (pILs) have been used to develop an all ionic liquid process for the dissolution and regeneration of silk (Goujon et al., 2012). Initially 5% w/w silk solutions in HFIP were regenerated using pILs based around the triethylammonium (TEA^+^) cation with differing anions: lactate (La^−^), triflate (OTf^−^), mesylate (OMs^−^) and dihydrogenphosphate (${\text{H}}_{\text{2}}{\text{PO}}_{4}^{-}$), each of which was in an 80%:20% w/w ratio of pIL to water. Fourier self-deconvolution of the FTIR spectrum was used to determine the secondary structure content of the silk foams regenerated with each of these coagulant solutions. The different anions were able to generate silk foams with very different structures. SEM showed a much more open and porous morphology for silk in [TEA][OMs] and a major peak at 22° shown in the XRD spectrum. Conversely, [TEA][H_2_PO_4_] showed a much tighter foam-like morphology and a single peak at 18.5° in the XRD spectrum, which is possibly a native-like structure (16.5°) but with a greater intersheet spacing. Focussing on the underlying secondary structure, [TEA][H_2_PO_4_] yielded the most native-like secondary structure with 55% β-sheet content (1,621–1,630 cm^−1^) and no α-helices (1,655–1,662 cm^−1^) whereas [TEA][OMs] yielded a silk foam with a large amount of α-helical structure (45%) as well as a large β-sheet content (50%).

Previous work with the amyloid peptide Aβ(1-40), best known for its implied role in Alzheimer's disease, foreshadowed the helix-inducing properties of [TEA][OMs] (Debeljuh et al., 2011). Through CD spectroscopy the secondary structure of Aβ(1-40) was monitored under varying [TEA][OMs] content. From 0 to 50% w/w [TEA][OMs] in water, the Aβ(1-40) retained its β-sheet secondary structure and ability to form fibrils. At 50–90% w/w the Aβ(1-40) changed to an α-helix secondary structure and no fibrils were formed. Finally at 90–100% w/w the Aβ(1-40) adopted the random coil conformation. Two potential hypotheses have been given for the mechanism by which pILs drive these conformational changes: the first is that the unique H-bonding network that pILs exhibit may drive proteins to preferentially form intermolecular bonds over intramolecular bonds. Alternatively, the microheterogeneity of pILs could be providing a membrane-like environment where a helical structure is preferred. Both of these hypotheses could help explain the preferential formation of α-helices by silk in [TEA][OMs].

For [TMG][La] (1,1,3,3-tetramethylguanidinium lactate) the general trend was that as water content increased, the β-sheet content fell, and the propensity for a microsphere morphology increased. Changes in the H-bonding network, alongside changes to the surface tension could drive the microsphere morphology. Yields decreased and coagulation time increased with increasing water content, consistent with amorphous silk being soluble in water. With this in mind an 80%:20% w/w [TMG][La]:water composition was used to coagulate silk from a 5% w/w solution in [C_4_C_1_Im][OAc]. The choice of both coagulant and solvent affects both morphology and secondary structure content. When HFIP was used to dissolve silk, a much finer foam structure was formed compared to [C_4_C_1_Im][OAc], containing larger, disordered voids. The secondary structure differed also with HFIP showing 55% β-sheet, 14% silk I structure, 12% α-helix and 9% β-turns, whereas using [C_4_C_1_Im][OAc] there was 64% β-sheet, 6% silk I, 27% α-helices and 2% β-turns. The demonstrated capability of pILs to alter protein conformation in a tunable sense is a compelling phenomenon and could provide good potential for generating precise morphology for future protein formulations.

## Enzymes

Potential applications for ionic liquids in the biotech-industry are numerous and an exhaustive list is not possible here. However, the limiting biocompatibility of these electrolytes has led to the emergence of very specific fields of implementation, whereby the advantage in overcoming issues of common chemical synthesis by deploying a reaction-selective biocatalyst outweighs the challenges of finding a suitable ionic liquid system. In this regard, the solubility of either the substrate or product can be identified as the primary driver that indicates use of an ionic liquid solvent may be valuable. The major focus areas of published research concerning biocatalysis in ionic liquids over the last decade have been in biofuel production, followed by the use as biosensors, and the production of enantiomerically-pure compounds (reviewed in Itoh, 2017b; Meyer et al., 2018). Protein stability in ionic liquids has been examined (Kumar and Venkatesu, 2014; Zhao, 2016), and provides part of the story, but cannot always accurately predict activity. A more thorough understanding of salt and enzyme interactions, including a classification of the impact of different anions and cations on enzyme activity, will enable applications that lie outside these dominating research fields, and is one of the rising areas of interest in the ionic liquid field. Some recent highlights for specific systems are provided here.

### Cellulases

Cellulases and their use in aqueous ionic liquids have one major, documented application: the saccharification of cellulose from various lignocellulose biomass sources for the production of biofuels. The treatment of the biomass with ionic liquids and the saccharification by the enzymes can be performed simultaneously, limited by the stability of the biocatalyst in such media. Pretreatment can be established through alkali, steam, acid or aqueous ammonia soaking, used for different substrates and exhibiting different yields (Ruiz et al., 2008; Cho et al., 2013; Govumoni et al., 2013; Maurelli et al., 2013). Pretreatment with ionic liquids dissolves lignin and hemicellulose by disruption of the hydrogen bonds of the cellulose to reduce crystallinity, thereby facilitating access for the hydrolytic enzymes (Tan and Macfarlane, 2010).

There is not a great variety of ionic liquids used for this process, as they need to have the ability to effectively compete with existing intermolecular H-bond interactions to separate the polymer chains (Table 4; Pinkert et al., 2010). The most usual cations are [C_4_C_1_Im] and [C_2_C_1_Im], and occasionally [AMIM] or [HEMA] (tris-(2-hydroxyethyl)-methylammonium). Similar to the situation with proteins, increasing the chain length of the imidazolium cation also leads to a decrease in dissolution of the cellulose (Kosan et al., 2007; Vitz et al., 2009; Cao et al., 2017). The anions [Cl], [OAc] and [BF_4_] have been most commonly investigated, followed by [Et_2_PO_4_] or [Me_2_PO_4_] and [MeSO_4_]. The hydrophobic [NTf_2_] ion is not capable of interacting strongly with the hydroxyl groups of the polymer chains. The H-bond acceptor ability and size of the anions primarily determine the ionic liquid dissolution ability (Pinkert et al., 2009), with a loose ranking of [SCN] < [Br] < dialkylphosphates < [OFo] ~ [OAc] ~ [Cl].

**Table 4:** Summary of selected cellulase reactions in ionic liquids.

**Ionic Liquid**	**Organism**	**Concentration range**	**Experimental outcome**	**References**
[AMIM][Cl]	*Hu*-CBH1	20–40% (v/v) aq, 2 M NaCl	~100–5% relative activity, respectively	Zhang et al., 2011
	Celluclast *T. reesei*	10% (v/v) aq	~ 25% relative residual activity (30 min)	Engel et al., 2010
	Cel5A from *T. tengcongensis*	2 M	50% relative residual activity after 5 h at room temperature	Liang et al., 2011
[AMIM][Me_2_PO_4_]	*T. reesei*	20, 50, and 100% (v/v) aq	34.36, 0.3, and 0% conversion, respectively, with ultrasonic heating pretreatment	Yang et al., 2010
[AEIM][Et_2_PO_4_]	*T. reesei*	20, 50, and 100% (v/v) aq	11.00, 0, and 0% conversion, respectively, with ultrasonic heating pretreatment	Yang et al., 2010
[C_1_C_1_Im][Me_2_PO_4_]	*T. reesei*	20, 50, 100% (v/v) aq	53.18, 1, and 0.3% conversion, respectively, with ultrasonic heating pretreatment	Yang et al., 2010
	Celluclast *T. reesei*	10% (v/v) aq	40% relative residual activity after 11 days	Engel et al., 2010
[C_1_C_1_Im][MeSO_4_]	α-galactosidase *Thermatoga maritimia*	0, 9, 27, and 45% (v/v)	42.7, 33, 6.2, 5.8 k_cat_/K_m_ s^−1^mM^−1^ respectively to the concentrations	Ferdjani et al., 2011
[C_1_C_1_MIm][MeSO_4_]	α-galactosidase *T. maritimia*	0-33% (v/v)	Similar activity profile to [C_1_C_1_Im][MeSO_4_] but less soluble	Ferdjani et al., 2011
[C_2_C_1_Im][Br]	Pseudoalteromonas sp. cellulase	1–20% (v/v) aq	115% relative activity at 5% (v/v)	Trivedi et al., 2013
[C_2_C_1_Im][CF_3_CO]	CelA2	30% (v/v) aq	506 mU/mg	Ilmberger et al., 2012, 2013
[C_2_C_1_Im][Cl]	*Tm*Bgl1A	200, 500 mM	1.32 × 10^3^ k_cat_/K_m_ (s^−1^ mM^−1^) at 200 mM	Kudou et al., 2014
	*S. cerevisiae* MT8-1	50–1000 mM	0.7 g/l ethanol production after 200 h	Nakashima et al., 2011
	*Hu*-CBH1	20% (v/v) aq 2 M NaCl	~120% relative activity	Zhang et al., 2011
[C_2_C_1_Im][Et_2_PO_4_]	*S. cerevisiae* MT8-1	50–1,000 mM	1.4 g/l ethanol produced after 200 h	Nakashima et al., 2011
	*T. reesei*	≤ 40% (v/v) aq	*In situ* one pot synthesis	Kamiya et al., 2008
	*T. reesei*	20, 50, and 100% (v/v) aq	2.18, 0, and 0% conversion at respective concentrations in ultrasonic heating pretreatment	Yang et al., 2010
[C_2_C_1_Im][EtSO_4_]	C. *rugosa* lipase	20% wt	Simulation revealed, that effect of altering enzyme charge is confined to short range (**<**1 nm) ordering of the IL	Burney et al., 2015
[C_2_C_1_Im][Me_2_PO_4_]	*T. reesei*	20, 50, and 100% (v/v) aq	48.14, 0.7, and 0% conversion at respective concentrations in ultrasonic heating pretreatment	Yang et al., 2010
[C_2_C_1_Im][MeSO_3_]	*Pseudoalteromonas sp*. cellulase	1–20% (v/v) aq	98% relative activity at 5% (v/v)	Trivedi et al., 2013
[C_2_C_1_Im][OAc]	*T. maritimia* endogluconase	5, 10, 15, and 20% (v/v) aq	52% decrease in specific activity at 15% (v/v)	Datta et al., 2010
	*T. viride* celullase	5, 10, 15, and 20% (v/v) aq	100% decrease in specific activity at 15% (v/v)	Datta et al., 2010
	*Pyrococcus horikoshii* endogluconase	5, 10, 15, and 20% (v/v) aq	5% decrease in specific activity at 15% (v/v)	Datta et al., 2010
	Cellulases from *A. terreus*	5, 10, 15, and 20% (v/v) aq	100% relative activity at 10% (v/v)	Gunny et al., 2014
	*Tm*Bgl1A	200 and 500 mM	3.15 × 10^3^ k_cat_/K_m_ (s^−1^ mM^−1^) at 200 mM	Kudou et al., 2014
	*S. cerevisiae* MT8-1	50–1,000 mM	1 g/l ethanol production after 200 h	Nakashima et al., 2011
	*Paenibacillus tarimensis*	20% (v/v) aq	90 and 80% relative activity at 80 and 50°C, respectively	Raddadi et al., 2013
	*Pseudoalteromonas sp*. cellulase	1–20% (v/v) aq	105% relative activity at 5% (v/v)	Trivedi et al., 2013
	β-glucosidase *T. reesei*	15 and 20% (w/v)	77 and 65% relative activity, respectively	Wang et al., 2011
	*Hu*-CBH1	20% (v/v) aq, 2 M NaCl	~ 100% relative activity	Zhang et al., 2011
[C_2_C_1_Im][OTf]	CelA2	30% (v/v) aq	54% relative activity	Ilmberger et al., 2012, 2013
	CelA3	30% (v/v) aq	68% relative activity	Ilmberger et al., 2012, 2013
	CelA *Thermatoga maritimia*	60% (v/v) aq	115% relative residual activity after 4 days	Ilmberger et al., 2012, 2013
[C_2_C_2_Im][Et_2_PO_4_]	*T. reesei*	20, 50, 100% (v/v) aq	18.55, 0.1, and 0% conversion at respective concentrations in ultrasonic heating pretreatment	Yang et al., 2010
[C_4_C_1_Im][BF4]	*Humicola insolens*	IL:Buffer 1:1	1.5 γ_c_ (g/l) after 6 h	Paljevac et al., 2006
[C_4_C_1_Im][Cl]	Cellulases from *A. terreus*	5, 10, 15, and 20% (v/v) aq	60% relative activity at 10% (v/v)	Gunny et al., 2014
	Cel5A from *T. tengcongensis*	40% (v/v) aq	80% relative residual activity after 5 h	Liang et al., 2011
	*Tm*Bgl1A	200 and 500 mM	1.89 × 10^3^ k_cat_/K_m_ (s^−1^ mM^−1^) at 200 mM	Kudou et al., 2014
	*T. reesei* engineered	0–5% (v/v) aq	~ 33, 18, and 16% conversion of 0.25 wt% lignin by succinylated, acetylated and wt enzyme, respectively	Nordwald et al., 2014
	*H. insolens*	IL:Buffer 1:1	0.3 γ_c_ (g/l) after 6 h	Paljevac et al., 2006
	*Paenibacillus tarimensis*	40% (v/v) aq	40 and 45% relative activity at 80 and 50°C, respectively	Raddadi et al., 2013
	*P. tarimensis*	20% (v/v) aq	75 and 70% relative activity at 80 and 50°C, respectively	Raddadi et al., 2013
	*Hu*-CBH1	20% (v/v) aq, 2 M NaCl	~105% relative activity	Zhang et al., 2011
	*Pseudoalteromonas sp*. cellulase	1–20% (v/v) aq	102% relative activity at 5% (v/v)	Trivedi et al., 2013
[C_4_C_1_Im][OAc]	Cellulases from *A. terreus*	5, 10, 15, and 20% (v/v) aq	60% relative activity at 10% (v/v)	Gunny et al., 2014
	*Tm*Bgl1A	200 and 500 mM	~23 U/mg at 200 mM (80°C)	Kudou et al., 2014
[C_4_C_1_Im][OTf]	89C12	30% (v/v) aq	111 mU/mg	Ilmberger et al., 2012, 2013
	*Pseudoalteromonas sp*. cellulase	1–20% (v/v) aq	94% relative activity at 5% (v/v)	Trivedi et al., 2013
[C_4_C_1_Im][PF_6_]	*H. insolens*	IL:Buffer 1:1	1.8 γ_c_ (g/l) after 6 h	Paljevac et al., 2006
[BMMIM][Cl]	CelA2	30% (v/v) aq	181 mU/mg	Ilmberger et al., 2012, 2013
	CelA3	60% (v/v) aq	79% relative residual activity after 4 days	Ilmberger et al., 2012, 2013
[C_4_C_1_Pyr][OTf]	CelA2	60% (v/v) aq	11% relative residual activity after 5 days	Ilmberger et al., 2012, 2013
	CelA84	30% (v/v) aq	8% relative activity	Ilmberger et al., 2012, 2013
	CelA84	60% (v/v) aq	81% relative residual activity after 4 days	Ilmberger et al., 2012, 2013
	CelA10	30% (v/v) aq	74% relative activity, 0.8% relative residual activity after 17 h	Pottkämper et al., 2009
	CelA24	30% (v/v) aq	2% relative activity	Pottkämper et al., 2009
	*Pseudoalteromonas sp*. cellulase	1–20% (v/v) aq	93% relative activity at 5% (v/v)	Trivedi et al., 2013
[Tris-(2-HOEt)-MAM][MeSO_4_]	*A. niger* endo-1,4-β-D-glucanase	0, 10, 50, and 99% (v/v) aq	Higher than average thermal stability; up to 140% relative activity at 75°C	Bose et al., 2012

A comprehensive overview of reports on enzymatic lignocellulose treatment is given in a publication by Wahlstroem and Suurnaeki (Wahlström and Suurnäkki, 2015). Early publications investigated *Trichoderma reesei* cellulase effectiveness in ionic liquids (Turner et al., 2003; Kamiya et al., 2008), and found very low enzymatic activity and stability. Following this, very interesting results were obtained by a study from Datta et al. (2010) on three structurally similar family-5-celullases obtained from the three different domains of life (Eukarya, Bacteria and Archaea; *Trichoderma viride* and thermophilic cellulases from *Thermogata maritime* and *Pyrococcus horikoshii*, respectively), which showed dramatically different activities in [C_2_C_1_Im][OAc] over a concentration range of 0–50% (v/v) (Datta et al., 2010). Relative specific activity was vastly different between all three. While *T. viride* cellulase activity had already declined to a third at 5% (v/v) IL, a linear trend of decreasing activity was observed for the cellulase from *T. maritima*, with about a third of the activity remaining at 20% (v/v). Activity stayed almost constant and equally high in comparison to the absence of ionic liquid for *P. horikoshii* cellulase in up to 20% (v/v) ionic liquid. However, by 50% (v/v) [C_2_C_1_Im][OAc], activity had completely declined for all enzymes, with only that from *P. horikoshii* showing the slightest activity.

Picking up on these differences Jaeger et al. (2015) conducted a MD study on those very same systems and found the impact of the ionic liquid derived in each case from individual local structural disturbances (Jaeger et al., 2015). Simulating a concentration of 0, 15, and 50%, they found that for *P. horikoshii* and *T. viride* only a few unstructured loops were displaced at both concentrations, while the secondary structure of the *T. maritima* cellulase is intensely disrupted. Moreover, results suggest that the secondary structure is more heavily disrupted at low to medium concentrations of around 15% and some kind of refolding or “reassuming of a prime-similar structure” occurs at 50%. The observation of refolding also held true for both of the other enzymes. RMSD values for these two enzymes indicated an even higher stability in 50% ionic liquid, compared to the buffer system. Their reported trajectories for the positively charged surface of *T. maritima* suggest that key salt bridges within the protein are broken and enable large structural changes. The RMSD values at 15% (v/v) suggest higher conformational stability, most likely due to the trapping of the enzyme in a less favorable conformation, reflected in the gradual decrease of activity in the study by Datta et al. (2010) For *T. viride* they suggest a likely competitive inhibition for substrate binding. Indeed, a recently published enzyme kinetic analysis of a commercially available cellulase cocktail finds competitive inhibition by [C_4_C_1_Im][Cl] is highly likely (Nemestóthy et al., 2017). In the case of the halophilic *P. horikoshii* cellulase, a possible deactivation at 50% (v/v) due to aggregation is suggested, because no major structural changes occur in the simulation (Jaeger et al., 2015).

Although many enzymes of other classes do not display their best activities in ionic liquids comprising chloride ions, a great number of cellulases seem to be particularly affected by [Cl] (Paljevac et al., 2006; Salvador et al., 2010; Ilmberger et al., 2013; Yoshimoto et al., 2013; Jaeger et al., 2015). For the highly positively surface-charged *T. maritima* celullase, which is also particularly enriched in α-helices, an investigative comparison between [C_4_C_1_Im] and [C_2_C_1_Im], in combination with [Cl] and [OAc], revealed that although conformational stability in low concentrations (0.2 M) of [C_4_C_1_Im][Cl] was extremely similar to the aqueous system with 10 mM phosphate buffer, a considerably decreased conformational stability in 0.2–0.5 M [C_4_C_1_Im][OAc] correlated with a considerably higher relative activity than for the other solvent systems, including the aqueous environment (Kudou et al., 2014). This relative activity was found to be highly temperature dependent and had an optimum at 80°C, which is unsurprising given it is a hyperthermophile-derived cellulase. Conformational restriction in [C_2_C_1_Im][OAc] manifested as an increased stability but to the point where the lowest activity was observed, as the active site could no longer be accessed effectively. These results imply that, as long as either catalytic residues are not displaced or the active center is not inhibited, a decreased conformational stability is rather preferable to a “rock-solid” conformation, where the active center is not flexible enough. The *k*_cat_/*K*_m_ values were also found to be considerably increased for [C_4_C_1_Im][OAc], indicating higher enzyme-substrate affinity (Kudou et al., 2014). These findings complement the observations by Jaeger et al. (2015) and Datta et al. (2010).

Halophilic cellulases appear to be promising biocatalysts and a recent patent out of the research on *Halorhabdus utahensis* cellulase Hu-CBH1 highlights this. This particular cellulase was found to display the same activity at 20% (v/v) [C_2_C_1_Im][OAc] and [AMIM][Cl] as in 2 M NaCl-buffer and activity was even slightly improved at 20% (v/v) [C_2_C_1_Im][Cl] (Zhang et al., 2011). The molecular basis for this improvement was postulated as due to the highly elevated negative surface charge brought about by a high proportions of Glu and Asp residues. There has been increasing interest in the investigation of halophilic celullases since Zhang's pioneering study (Ilmberger et al., 2012; Raddadi et al., 2013; Gunny et al., 2014; Nordwald et al., 2014). Ilmberger et al. (2012) used a concentration of 30% (v/v) of [C_4_C_1_Im][Cl] and [BMMIM][Cl] (1-butyl-2,3- diemthylimidazolium), which yielded moderate specific activities from newly isolated, moderately thermophilic and halotolerant enzymes CelA2 and CelA3 from a biogas plant.

Raddadi et al. (2013) assayed a crude lysate of *Paenibacillus tarimensis* and demonstrated a clear correlation between temperature and salt concentration, which was also found in other studies regarding halophilic enzymes (Zhang et al., 2011; Gunny et al., 2014). While an increase of salt concentration in aqueous buffer systems leads, at low-moderate temperatures, to a decrease in activity, at high temperatures the opposite is found. However, this does not apply to ionic liquid concentrations. Mixtures of aqueous solution of 20 and 40% (v/v) [C_4_C_1_Im][Cl] without NaCl in the buffer showed lower activity for the higher concentration at higher temperatures (Raddadi et al., 2013). Interestingly, if the buffer-ionic liquid solution contained an additional 5 M NaCl, a slight increase in activity at higher temperatures was effected at 20% (v/v) [C_4_C_1_Im][Cl] and [C_2_C_1_Im][OAc]. The sodium ions are likely the essential mitigators, coordinating to the negatively charged surface residues. Systems with [C_2_C_1_Im][OAc] displayed overall the highest activity with ~90% relative activity. Optimum activity for an *Aspergillus terreus*-derived cellulase was reached in low to medium concentrations of 10% (v/v) [C_2_C_1_Im][OAc] with almost the same relative activity compared to the buffer system (Gunny et al., 2014). This study also found a similar salt-mitigating temperature dependence as per the Raddadi study.

Finally, another study examining *T. reesei* cellulase once more, modified the enzyme surface charge by succinylation and acylation, finding a moderate improvement for succinylation and a slight negative effect for acylation in [C_4_C_1_Im][Cl] (Nordwald et al., 2014). This adverse effect of acylation in comparison to natively, negatively-charged enzymes could be explained by, and at the same time verify, the theory that negatively-charged surface residues are not randomly placed, but rather “strategically” favorable (Madern et al., 2000). This placement acts either to have these residues repel and lend the protein more flexibility; or to coordinate positively charged ions to secondary structural regions of interest, e.g., to α-helices, which seem to be more affected by salt than β-sheets; or a combination of both.

In conclusion, the studies of cellulases in ionic liquids have demonstrated that small positively charged ions (Na^+^, K^+^) seem to be essential for structural integrity, at least to enzymes that possess a negatively-charged surface. If the surface charge is positive, symmetrical, smallish, negatively-charged ions (Cl^−^) are attracted to the surface and disrupt the secondary structure substantially, with the coordinating strength of the anion to the surface depending on its H-bond acceptor ability. This disruption does not automatically lead to a major disturbance of the active center, unless inhibition by the ionic liquid ions occurs. Upon increasing ionic liquid concentrations, overall enzyme structure can get trapped by the charged molecules; this applies already at low to moderate concentrations for mesophilic proteins and at higher concentrations of ionic liquid for halophilic proteins. In the latter case, the negatively charged surface residues most likely repel the negatively charged ions of the solution.

### Alcohol Dehydrogenases

Alcohol dehydrogenases (ADHs) are valuable biocatalysts due to their enantioselective reactions with prochiral ketones and alcohols. Productivity for a broad range of substrates, however, is limited by the incompatibility of the solubility of the required redox cofactor NAD(P)H in non-aqueous media and sparingly water-soluble ketone substrates. This makes ionic liquids an attractive medium for reaction, where compatibility with the enzyme can be established.

A range of ionic liquids, primarily based on either imidazolium or ammonium cations (Table 5), have been employed with ADHs. The mechanism of impact of ionic liquids on ADH can be interpreted through the prism of three ionic liquid classifications: (a) hydrophilic ionic liquids (b) water-immiscible ionic liquids, which drive the partition coefficients of substrates and products in favor of the enzyme without direct interaction and (c) water-miscible, amphiphilic ionic liquids, which provide H-bonding through the hydrophilic part and substrate solubility within their alkyl-tail-pockets.

**Table 5:** Summary of ADH reactions in selected ionic liquids.

**Ionic Liquid**	**Enzyme**	**Concentration ranges**	**Experimental outcomes**	**References**
[C_4_C_1_Im][PF_6_]	YADH	IL:EtOH:H_2_O	Catalytic activities between 0.7 and 11 mol L^−1^ min^−1^ recorded, depending on (up to 0.55 IL)	Zhang et al., 2011
	YADH	IL:H_2_O:TritonX-100	Catalytic activity 51 mol L^−1^ min^−1^ with ratios 0.1:0.3:0.6, respectively.	Zhang et al., 2011
	HL-ADH	0.025–0.4 g/ml	Enhanced activity (up to 145% at 0.025 g/ml) dropping to 95% and then < 50% at 0.075 and 0.15 g/ml and above, respectively. Half-life 1.6 h with residual activity at 50 h.	Shi et al., 2006
	RE-ADH	10% v/v (aq)	Conversion rate 98.5%; Activity 28% Half-life 135 h.	Hussain et al., 2008
	*R*. *ruber* ADH-A	20, 50, and 80% v/v (aq)	Conversion rates ~30, 20, and 15%, respectively	De Gonzalo et al., 2007
[C_4_C_1_Im][NTf_2_]	LB-ADH	Biphasic with 50 mM phosphate buffer	88% conversion; 61% conversion in MTBE/buffer	Eckstein et al., 2004
	W110A TE-ADH	50% v/v (aq)	Conversion 52% - >99% with a selection of substrates	Musa et al., 2008
	*R*. *ruber* ADH-A	20, 50, and 80% v/v (aq)	Conversion rates ~25, 10, 5%, respectively	De Gonzalo et al., 2007
[C_4_C_1_Im][Cl]	HL-ADH	0.025–0.4 g/ml	Enhanced activity (up to 155% at 0.05 g/ml) dropping to 90% and then < 50% at 0.2 and 0.4 g/ml, respectively. Half-life 14.5 h with residual activity at 50 h.	Shi et al., 2006
	YADH	0.01–0.6 M	98–20%, respectively; enzyme parameters.	Dabirmanesh et al., 2011, 2012
[C_4_C_1_Im][Br]	HL-ADH	0.025–0.4 g/ml	Enhanced activity (up to 185% at 0.05 g/ml) dropping to 90% and then < 50% at 0.15 and 0.4 g/ml, respectively. Half-life 5.3 h with residual activity at 50 h.	Shi et al., 2006
[C_4_C_1_Im][BF_4_]	HL-ADH	0.025–0.4 g/ml	Activity ~30% at 0.025 g/ml dropping to ~0% at 0.075 g/ml. Half-life < 0.5 h.	Shi et al., 2006
	RE-ADH	10% v/v (aq)	Conversion rate 99.5%; Activity 38% Half-life 82 h.	Hussain et al., 2008
	W110A TE-ADH	50% v/v (aq)	Conversion 40–96% with a selection of substrates	Musa et al., 2008
	YADH	0.01–0.4 M	95–20%, respectively; enzyme parameters.	Dabirmanesh et al., 2011, 2012
[C_4_C_1_Im][OAc]	*R*. *ruber* ADH-A	20, 50, and 80% v/v (aq)	Conversion rates ~40, 10, and 0%, respectively	De Gonzalo et al., 2007
[C_4_C_1_Im][OTf]	HL-ADH	0.025–0.4 g/ml	Activity ~25% between 0.025 and 0.1 g/ml dropping to ~0% at 0.15 g/ml. Half-life 1.5 h.	Shi et al., 2006
[C_2_C_1_Im][OAc]	*R. ruber* ADH-A	20, 50, and 80% v/v (aq)	Conversion rates ~ 45, 2, and 0%, respectively	De Gonzalo et al., 2007
[C_2_C_1_Im][Cl]	HL-ADH	0.025–0.4 g/ml	Enhanced activity (up to 155% at 0.025 g/ml) dropping to 95% and then < 50% at 0.075 and 0.4 g/ml, respectively.	Shi et al., 2006
[C_2_C_1_Im][Et_2_PO_4_]	LB-ADH	10% v/v (aq)	Activity ~ 5%	Kohlmann et al., 2011
[C_2_C_1_Im][Me_2_PO_4_]	LB-ADH	10% v/v (aq)	Activity ~ 80%	Kohlmann et al., 2011
[C_2_C_1_Im][EtSO_4_]	RE-ADH	10% v/v (aq)	Half-life 147 h.	Hussain et al., 2008
[C_2_C_1_Im][MeSO_3_]	*R*. *ruber* ADH-A	20, 50, and 80% v/v (aq)	Conversion rates ~5, 0, and 0%, respectively	De Gonzalo et al., 2007
	LB-ADH	10% v/v (aq)	Activity ~ 105%	Kohlmann et al., 2011
[C_2_C_1_Im][MDEGSO_4_]	RE-ADH	10% v/v (aq)	Half-life 22 h.	Hussain et al., 2008
	LB-ADH	10% v/v (aq)	Activity ~ 70%	Kohlmann et al., 2011
[C_2_C_1_Im][OTs]	RE-ADH	10% v/v (aq)	Conversion rate 89%; Activity 51% Half-life 45 h.	Hussain et al., 2008
[C_1_C_1_Im][Me_2_PO_4_]	RE-ADH	10% v/v (aq)	Conversion rate 38.5%; Activity 66% Half-life 182 h.	Hussain et al., 2008
[C_1_Im][BF_4_]	YADH	0.01–0.6 M	85–0%, respectively with zero activity from 0.4 M; enzyme parameters.	Dabirmanesh et al., 2011, 2012
[C_1_Im][Cl]	YADH	0.01–0.2 M	80–0%, respectively; enzyme parameters.	Dabirmanesh et al., 2011, 2012
[3-HOMeC_2_Py][EtSO_4_]	RE-ADH	10% v/v (aq)	Conversion rate 100%; Half-life 266 h.	Hussain et al., 2008
[C_4_C_1_Pyr][NTf_2_]	RE-ADH	10% v/v (aq)	Activity 43%; Half-life 144 h.	Hussain et al., 2008
AmmoEng™ 100^*^	*R*. *ruber* ADH-A	70, 80, and 90%	Conversion rates 77.5, 73.1, and 27.0%, respectively	De Gonzalo et al., 2007
	LB-ADH	10% v/v (aq)	Activity ~ 150%	Kohlmann et al., 2011
AmmoEng™ 101^*^	*R*. *ruber* ADH-A	70, 80, and 90%	Conversion rates 78.2, 77.4, and 28.3%, respectively	De Gonzalo et al., 2007
	LB-ADH	10% v/v (aq)	Activity ~ 180%	Kohlmann et al., 2011
AmmoEng™ 102^*^	RE-ADH	10% v/v (aq)	Conversion rate 100%; Activity 9.4%; Half-life 12 h.	Hussain et al., 2008
	*R*. *ruber* ADH-A	70, 80, and 90%	Conversion rates 69.7, 67.1, and 7.5%, respectively	De Gonzalo et al., 2007
	LB-ADH	10% v/v (aq)	Activity ~ 110%	Kohlmann et al., 2011
AmmoEng™ 110^*^	RE-ADH	10% v/v (aq)	Half-life 77 h.	Hussain et al., 2008
AmmoEng™ 112^*^	LB-ADH	10% v/v (aq)	Activity ~ 100%	Kohlmann et al., 2011
AmmoEng™ 120	RE-ADH	10% v/v (aq)	Half-life 40 h.	Hussain et al., 2008
AmmoEng™ 140	LB-ADH	10% v/v (aq)	Activity ~ 90%	Kohlmann et al., 2011
[N_8, 8, 8, 1_][NTf_2_]	*R*. *ruber* ADH-A	20, 50, and 80% v/v (aq)	Conversion rates ~40, 20, and 10%, respectively	De Gonzalo et al., 2007
[Tris-(2-HOEt)-MAM][MeSO_4_]	LB-ADH	10% v/v (aq)	Activity ~ 120%	Kohlmann et al., 2011
	R. *ruber* ADH-A	70, 80, and 90%	Conversion rates 79, 76.4, and 65.3%, respectively	De Gonzalo et al., 2007

**Figure 2:**
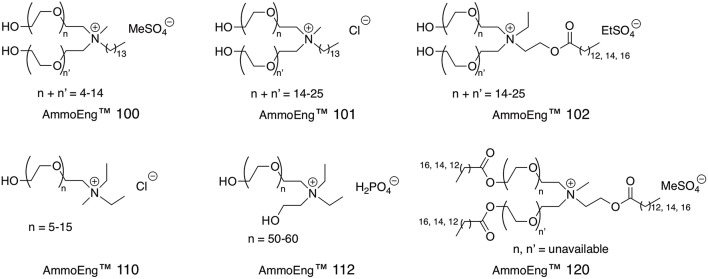
The AmmoEng™ series of ionic liquids.

*MDEGSO_4_, 2-(2-methoxyethoxy)ethylsulfate*;

* *REACH registered*.

- **Hydrophilic ionic liquids**
  - Ion effects
    The impact of hydrophilic ions, such as Me_4_N, choline, and imidazolium cations, and Cl, Br, BF_4_, PF_6_, OTf, Me_2_PO_4_, Et_2_PO_4_, MeSO_3_ anions, has been examined for a number of ADH systems, with the caveat that PF_6_ or BF_4_ ions can be hydrolysed in aqueous solutions. While PF_6_ hydrolysis is mainly promoted only under acidic conditions so that the PF_6_ ion can be suitable for use at moderate temperatures in aqueous solutions, activity in BF_4_ might not be fully attributed to the biocatalyst, as it is not stable in water and will hydrolyse readily (Freire et al., 2010). The ionic liquids [C_2_C_1_Im][Cl], [C_4_C_1_Im][PF_6_], [C_4_C_1_Im][Cl] and [C_4_C_1_Im][Br] were able to enhance activity of HlADH at low concentrations between 0.05 and 0.075 M, when compared to pure buffer systems (Shi et al., 2006). This finding may hold true for many more small-molecule ions, but unfortunately not many ADH studies in ionic liquids apply alternatives to imidazolium cations or examine such low concentrations (see Table 5). Comparability of results in this field is also challenging as reported results vary between conversion rates, yields and residual activity. Regardless, conversion rates and half-life of enzymes seem to show considerable improvements upon addition of aqueous ionic liquids, relative to buffer solutions at low to moderate concentrations. In particular, improvements have been seen for [C_4_C_1_Im][OAc] and [C_2_C_1_Im][OAc] at 20% (v/v) (De Gonzalo et al., 2007), for [C_2_C_1_Im][MDEGSO_4_], [C_2_C_1_Im][MeSO_3_], and [C_1_C_1_Im][Me_2_PO_4_] at 10% (v/v) with respect to conversion rates (Kohlmann et al., 2011), and for [EMP][ES], [C_4_C_1_Im][BF_4_], and [C_2_C_1_Im][OTs] at 10% (v/v) with respect to half-life stability (Hussain et al., 2008). In contrast, imidazole and imidazolium derivatives, because of their similarity in structure to parts of the cofactor NADP^+^ (Dabirmanesh et al., 2012), have been reported as exhibiting competitive inhibition of the active center of ADHs (Zhang et al., 2008), and with smaller alkyl chains lengths for these imidazolium-based ionic liquids the inhibitory effect increases (Dabirmanesh et al., 2012).
    Chloride-ion based ionic liquids do not act as a challenge to ADH-systems. YADH assayed in [C_4_C_1_Im][Cl] lead to a slight decrease in activity (~95%) at very low concentrations (~0.01 M), but activity (~20% remaining) was maintained up to a concentration of ~0.6 M ionic liquid, while for [C_1_C_1_Im][Cl] only 80% remaining activity was observed at 0.01 M and activity had completely declined at ~ 0.2 M (Dabirmanesh et al., 2011). For HlADH assayed in [C_4_C_1_Im][Cl] a maximum relative activity of 150% was observed at 0.075 M and decreased slowly to 25% at 0.4 M ionic liquid concentration. Use of [C_2_C_1_Im][Cl] lead to a maximum relative activity of 150% at 0.05 M, but activity values were ~50% less on average at higher concentrations (0.1–0.3 M) than for [C_4_C_1_Im][Cl] (Shi et al., 2006). However, activity ended up being about the same at 0.4 M in both ionic liquids, which was the highest concentration assayed. Of the other hydrophilic ionic liquid systems that have been assayed, [C_2_C_1_Im][Et_2_PO_4_] is strikingly detrimental to the enzymatic activity of *L. brevis* ADH with only 5% relative activity left at 10% (v/v) for the reduction of 2-octanone. This contrasts with [C_1_C_1_Im][Me_2_PO_4_], with 85% relative activity, and is comparable to the activity of YADH found for [C_1_C_1_Im][Cl] (above), and [C_2_C_1_Im][MeSO_3_] with 105% relative activity (Kohlmann et al., 2011).
    Simulation studies have been able to shed light on the specific interactions that may be at play in controlling activity. Ionic liquid ions with low charge density (most cations) do not tend to exhibit any preference of vicinity on the protein surface, since coulombic forces for these ions are less dominant due to delocalisation and are competitive with the van der Waals interactions of the alkyl chains. Thus, these ions tend to be more mobile on the protein surface, than those ions with high charge density (most anions) (Tomé et al., 2012; Lim and Klähn, 2018), and this gives a rationale for the relatively more significant impact ascribed ionic-liquid anions. At all concentrations, the cations with their alkyl chains are able to contact like-charged-ions, but at low concentrations, since there are only a few about, they will, as single ions or small clusters, be able to closely associate to the protein surface.
    The shorter the alkyl chain, the smaller are the polar domains that can interact with other species meaning, [C_1_C_1_Im] or [C_2_C_1_Im] can associate more closely and individually onto the surface of the protein and more easily into the binding pocket, than, for example C_4_C_1_Im. The peak in activity at very low concentrations of ionic liquid, and the consequent decrease in activity at increasing concentrations of ionic liquid may be attributed to the different numbers of cations being able to aggregate on the protein surface. As the cations continue to coordinate to the protein surface at increasing concentrations (Lim and Klähn, 2018), the ions form ever bigger patches, that are still small enough to amass onto the protein surface, but with the increasing patch-size an increased rigidity of the protein is conferred. The discrepancy of ~50% less activity between [C_4_C_1_Im] and [C_2_C_1_Im] at medium range concentrations (0.1–0.3) underlines the different interactions of the ions with the protein surface, as [C_2_C_1_Im] having shorter chains will be able to associate more closely overall. This close association allows an optimum effect on the flexibility (and activity) of the protein at a slightly lower concentration (0.05 M), than [C_4_C_1_Im] with 0.075 M, and at the same time having a much higher impact on constraining the protein flexibility at medium concentrations. At low ionic strength, the ring and tail of the imidazolium ions are equally often in contact with the protein surface, whereby the tail mainly has a steric interaction (fitting into clefts on the surface) and the ring has a mostly charged interaction, which becomes more pronounced and dominant at higher ionic strengths (Haberler et al., 2011). In comparison, high-charge density ions, if located on the surface, preferentially reside at positions with oppositely charged amino acids and hardly associate with like-ions. At low ionic liquid concentrations the number of low charge density ions populating the protein surface is far greater than the number of high charge density ions, which prefer the hydration of water molecules in the bulk phase (Haberler et al., 2011), leading to asymmetric influence at different concentrations on the protein.
    Anions with high charge density, being dispersed in bulk water at low concentrations, may strip off a small number of tightly bound, structuring water molecules from the protein surface, leading to an increase in entropy and hence more flexibility and activity of the enzyme, up to the maximum point, upon thereafter the disruption of the secondary structure and subsequent rigidity of the protein (as seen for cellulases) becomes detrimental. This can affect the binding pocket significantly, as was proposed by the study on the enantioselectivity of *Thermoanaerobacter ethanolicus* ADH (Musa et al., 2008). In aqueous solution, the binding pocket of *T. ethanolicus* ADH includes a bound, ordered water molecule, which is displaced when a bulky (*R*)-substrate binds, yielding a substantial entropy increase (Heiss et al., 2001). For smaller substrates there is no enantioselective conversion in aqueous solution because they are unable to displace the water molecule. Addition of ~50% (v/v) of the hydrolysable ionic liquid [C_4_C_1_Im][BF_4_] was able to facilitate the asymmetric reduction of small phenyl-ring containing ketones, with this result suggested as indicating the expulsion of the tightly bound, structured water molecule by the ionic liquid (Musa et al., 2008). A similar result was obtained using ~50% (v/v) of the water immiscible [C_4_C_1_Im][NTf_2_], where the lower concentration of substrate in the aqueous phase was used to account for the increase in enantioselectivity, highlighting that a more microscopic view of the reaction may be required to fully interpret the role of the ions.
  - Water-effects
    The change in activity seen for ADHs, spiking around one mole fraction of water (χ_H2O_ ~ 0.9) highlights the finding of a “magic point,” reported in a computational solvation study of a small zinc-finger protein in aqueous ionic liquids (Haberler et al., 2011). This point hints at a change in the organization of the ions and water molecules at *ca*. 0.075 M ionic liquid-aqueous mixture, which appears to correlate with a transition from dipolar screening (achieved by small water molecules intercalating between protein residues and attenuating residue-residue interactions) to charge screening (by ion-charge interactions) of electrostatic forces. At this transition point the protein is at its highest total energy and lowest level of ordering of the secondary structure (Haberler and Steinhauser, 2011; Haberler et al., 2011, 2012). At comparably high ionic strengths the mean residence times of ions, especially anions, on the protein surface, i.e., their interaction with specific residues, is raised from picoseconds to nanoseconds (Haberler et al., 2011), and a weakening effect on the binding affinity of the substrate to the enzymes is observed (Dabirmanesh et al., 2011, 2012).
    Furthermore, at increasing concentrations of ionic liquids, simulation studies show that the water molecules change their behavior significantly (Gehrke et al., 2018). At relatively low mole fractions of water (0.38) water molecules tend to form larger clusters of around 12 molecules, and they are apparently present either as single molecules or on-average as dimers at higher mole fractions of ~ 0.82 (Gehrke et al., 2018). Here, the underlying structuring force of the van der Waals-induced, tetrahedral-coordinating water molecules becomes replaced by an ionicity driven network, which removes interstitial water molecules as the number of high-charge density ions increases in the bulk. This is in agreement with the observation of an overall dielectric decrement at reduced mole fractions of water, which is especially consequential in the first hydration shell (Haberler et al., 2012).
- **Water-immiscible ionic liquids (biphasic systems)**
  The water-immiscible ionic liquid [C_4_C_1_Im][NTf_2_] was one of the first ionic liquids successfully implemented for improving the enantioselective reduction of ketones with ADH (Eckstein et al., 2004), and since then biocatalysis in multiphasic ionic liquid reaction systems has advanced to show the potential for industrial scale applications of these systems (Meyer et al., 2018). In comparison with hydrophilic ionic liquids, for which a maximum tolerance of around 20% (v/v) of [C_2_C_1_Im][OAc] (the best candidate with regards to conversion rates), was found, biphasic systems can provide meaningful application maxima of ~80% (v/v) and still retain the enzyme's hydration shell (De Gonzalo et al., 2007). Since the enzyme and the cofactor are in this case “immobilized” in the aqueous phase, it is the partition coefficient of the substrate and co-substrate in the ionic liquid and aqueous phase that drives the thermodynamics of the reaction. In this regard, these biphasic systems are optimal replacements for conventional organic co-solvents as they can be ion-specifically selected to facilitate different partition coefficients for particular substrates and co-substrates, as has been shown for 1-hexyloxymethyl-3-methyl-imidazolium and 1, 3-dihexyloxymethyl-imidazolium based ionic liquids with alcohols, water, ketones and hydrocarbons (Domanska and Marciniak, 2007). In particular, programmes such as COSMO-RS have been successfully validated for predicting phase equilibria for such systems containing imidazolium and pyridinium-based ionic liquids (Freire et al., 2007).
- **Water-miscible, amphiphilic ionic liquids**
  Hydroxyl-functionalised ionic liquids, including the AmmoEng™ series of task-specific ionic liquids (Figure 2), have been shown to enhance the activity of ADHs even at very high concentrations of up to 90% (v/v) ionic liquid content (De Gonzalo et al., 2007), with the optimal concentration dependant on the experimental set-up. Up to 90% (v/v) is tolerated when whole cell-biocatalysts or lysates thereof are applied (De Gonzalo et al., 2007), whilst for purified enzyme, solution concentrations of 10% (v/v) AmmoEng™ 101 were shown to enhance the relative activity of *L. brevis* ADH by 180% (Kohlmann et al., 2011). A major advantage of the use of these amphiphilic ionic liquids is the much higher than usual solubility of difficult substrates, as has been demonstrated for, for example, 4'-Br-2,2,2-trifluoroacetophenone and 6-bromo-β-tetralone (Hussain et al., 2008). *Rhodococcus erythropolis* ADH and GDH 103 did not show any residual activity for those substrates when used in buffer, but GDH 103 showed 65% (w/w) residual activity in 10% (v/v) AmmoEng™ 102. Both ADH and GDH 103 enzymes showed 50% (w/w) residual activity in the water-immiscible ionic liquid [C_4_C_1_Pyr][NTf_2_] (Hussain et al., 2008). The amphiphilic oligoether-based ionic liquids AmmoEng™ 100 and 101, with a 14-carbon coconut oil-derived group, were found to either impart the most activity or increase conversion by 150 and 180%, respectively (De Gonzalo et al., 2007; Kohlmann et al., 2011), with the main difference being the anion [MeSO_4_ (100) and Cl (101)]. AmmoEng™ 102, which has an 18-carbon tallow-derived group, a 5-fold longer oligio(ether-) chain than AmmoEng™ 101 and a greater ability to absorb water due to the higher ether content (Ribot et al., 2012), was found to increase activity of *L. brevis* ADH by 110%. In AmmoEng™ 140 only a 90% increase in activity was observed.

### Lipases

Lipase biotransformations have significant application, and this is reflected in the ionic liquid field by a substantial literature (Itoh et al., 2002; Zhao et al., 2010a, 2018; Latif et al., 2014; Kim et al., 2016; Carvalho et al., 2018; Itoh, 2018; Lisboa et al., 2018; Park et al., 2018; Zhong et al., 2018). As such, lipase-catalyzed biotransformations have been the topic of recent reviews, covering enantioselective transesterification, biofuel production and polymer synthesis in ionic liquids (Itoh, 2017a; Elgharbawy et al., 2018), and ionic liquid-mediated activation of lipase-catalyzed reactions (Itoh, 2018). Further, for specific lipase-catalyzed reactions there has been a more extensive fine tuning of the use of ionic liquids than for other enzymatic systems. A prominent example is the deployment of [BDMIM] instead of [C_4_C_1_Im] in the prevention of oligomerisation of acetaldehyde, where the polymer product inhibits the reaction after repeated cycles (Itoh et al., 2002).

Dynamic kinetic resolution (DKR) can be enhanced through the use of lipase in ionic liquids. The polymerisable ionic liquid [VEIm][Br] (1-vinyl-3-ethylimidazolium bromide) was used to immobilize the lipase CalB as a hydrogel, with application for the kinetic resolution of 1-phenylethanol through transesterification (Grollmisch et al., 2018). Upon immobilization, the conversion after 5 h was nearly 15% higher and only the (*R*)-product was formed with an ee of > 99%, whilst the unreacted (*S*)-educt accumulated in the reaction mixture with twice the ee relative to the non-immobilized system (Grollmisch et al., 2018). Another immobilization study applying CalB focused on the catalytic selectivity toward diacylglycerols production (Zhong et al., 2018), where the immobilization substrate was modified with one of either [C_4_C_1_Im], [C_4_C_4_Im] or [C_4_C_8_Im]-like cations combined with either the [BF_4_] or [PF_6_] anion. Here the [BF_4_] anion-modified material improved activities, whereas those coupled with the [PF_6_] anion did not. Fish oil production has also been attempted with imidazolium-based ionic liquid–immobilized lipase systems utilizing the two hydrolysable ionic liquids [C_4_C_1_Im][BF_4_] and [C_4_C_1_Im][PF_6_], and the hydrophobic ionic liquid [C_4_C_1_Im][NTf_2_] (Fu et al., 2018).

Revisiting the *Candida rugos*a lipase (CRL) for the enantioselective hydrolysis of racemic ketoprofen ethyl ester, Park et al. (2018) assessed six different [C_4_C_1_Im] derivatives, finding 5% (v/v) solution of [C_4_C_1_Im][MeSO_4_] afforded highest conversion (47.3% after 23 h) and enantiomeric excess (ee_p_ > 99%), but observed long term instability for this ionic liquid mixture (Park et al., 2018). A 20% (v/v) solution of [C_4_C_1_Im][PF_6_] yielded a conversion of 48% with 96.9% ee_p_ and the enzyme showed much higher long term stability. With the exception of [MeSO_4_], for which an acid effect was attributed to the improved activity, decreasing H-bond basicity of the ionic liquid anion correlated with an increase in enantioselectivity (Park et al., 2018).

Biodiesel production with lipase has had well-designed early strategies (Arai et al., 2010; Zhao et al., 2010a; Lozano et al., 2013), although inactivation of the enzyme through the effect of ethanol during transesterification is still an obstacle preventing large scale applications. This challenge may soon be overcome, with an effective model reaction to describe the inactivation of the lipase Novozyme 435 recently being proposed, and a circulating feed mixture being introduced into the reactor design to remove the by-product glycerine (Endo et al., 2018). The immobilization of *Burkholderia cepacia* lipase onto silica xerogel with protic ionic liquid (*N*-methylmonoethanolamine pentanoate) and in-depth characterization of the activity and operational stability could afford appropriate catalysts for such a circulating reactor (Carvalho et al., 2018). Experimental data of the physical adsorption of *B. cepacia* lipase on an aerogel-ionic liquid system based on [N_1, 1, 1, 18_][NTf_2_], with an isotherm model fitted to the data, provides an alternative, characterized immobilization system (Lisboa et al., 2018).

Simulations of lipases in ionic liquids have been used to elucidate surface interactions between the enzyme and the cations and anions. Combining experimental and simulation techniques, chloride anions of [C_4_C_1_Im][Cl] were identified as inducing a conformational switch between an α-helix and turn in CalB, with a specific interaction with Lys290 leading to a narrow cavity entrance and directly reducing the activity (Kim et al., 2014). The transesterification of butyl alcohol was carried out in pure [C_4_C_1_Im][OTf], [C_4_C_1_Im][Cl], tert-butanol and 0.3 M NaCl, and simulation in these solvents showed that in water the cavity to the catalytic center can be opened and closed, in tert-butanol and [C_4_C_1_Im][OTf] it resumes the open conformation, but for [C_4_C_1_Im][Cl] the cavity is closed. The electrostatic energy between the anion and the enzyme was proposed as the driving force for the change in conformation. A follow-up study in 2016, using all-atom MD simulations to look at cation effects by varying the lengths of the alkyl tails of Imidazolium ions, found that medium length (butyl–hexyl) alkyl chains disrupt the catalytic activity the least, while [C_2_C_1_Im], having a high ion coordination number, prevents the interaction between the key lysine and isoleucine residues controlling the cavity conformation. Longer alkyl chains (e.g., octyl) exhibit strong hydrophobic interactions with a nearby leucine, which leads to the complete loss of the secondary structure of the α-helix. Interaction of [C_8_C_1_Im] with the hydrophobic leucine next to the catalytic center induces an opening up of the pocket and allows [NTf_2_] to locate and interact with the catalytic residues (Kim et al., 2016).

The role of water is important in understanding the action of aqueous ionic liquids on enzyme activity. Simulations of *Candida antarctica* lipase B (CalB) and CRL in the [C_4_C_1_Im]-based ionic liquid systems, with the counter-ions [PF_6_], [BF_4_], [Cl], [OTf], and [NTf_2_], respectively, found a bell-shaped dependency of enzyme structural deviations (RMSD values) mirroring water-content (Latif et al., 2014), as had been previously reported for organic solvents and other ionic liquid-enzyme systems (Laszlo and Compton, 2001; Micaêlo and Soares, 2008). Non-localized water molecules were stripped off the enzyme surface with the degree of observed removal related to the solubility and hydrophobicity of the anions (Latif et al., 2014). Ions with high charge density (most anions studied) are preferentially dispersed in bulk water at low concentrations (Haberler and Steinhauser, 2011; Haberler et al., 2012), and are those that strip the non-localized water molecules. Chloride ions in aqueous solution require six water molecules to form a first hydration shell; in a study of the behavior of [C_8_C_1_Im][Cl] at both the interface and in bulk solution, the calculated water to ion pair molar fraction was found to be 3.6 at ~20 wt%, meaning that the chloride ion was preferentially surrounded by one cation and 3-4 water molecules (Cheng et al., 2018). This structuring, and rearrangement of anionic coordination sphere could also have implications for the nature of the interaction of ions with a protein surface at different water contents. In addition, different surface topologies could also produce differential hydrogen-bond and ion-interaction dynamics (Dahanayake and Mitchell-Koch, 2018), leading to localized effects, individual to each protein and for particular solvation conditions.

### Laccases

Solubilizing wood-derived biomass really dictates the need for ionic liquids, in the same way as these solvents are required for cellulases to access the cellulose substrate. Protein engineering to enhance activity and stability of laccase in the required ionic liquids is an attractive approach, as engineering of the ionic liquid-based reaction media can be challenging within the constraints of lignin solubility. Alternatively, two-phase systems have been successfully utilized (Xue et al., 2011).

Liu et al. (2013) reported the directed evolution of a laccase from *Trametes versicolor*, which increased its stability and its activity by 4.5-fold in 15% (v/v) [C_2_C_1_Im][EtSO_4_], relative to buffer. Upon alteration of two residues, activity was also increased in buffer, but only by 3.5-fold. Fungal laccases comprise of three domains, whereby domains 2 and 3 are connected *via* an exceptionally long loop (Bertrand et al., 2002). A synergistic substitution of a residue in the connecting loop and a residue in the catalytic center led to an increase in hydrophobicity in the loop region, increasing stability and providing better access to the catalytic center of the protein (Liu et al., 2013). The increase in hydrophobicity is proposed to enhance interactions between domains 2 and 3 under the augmented ionicity of the ionic liquid solution. Based on the identified importance of the changes in hydrophobicity of the domain-connecting loop, a follow-up study focused on synergistic substitutions of alanine residues to (mainly) charged residues in the loop region (Wallraf et al., 2018). The improved rigidity imparted by removing the flexible alanine residues, afforded a particular synergistic improvement in activity at a concentration of 5–15% (v/v) [C_2_C_1_Im][EtSO_4_] (Wallraf et al., 2018). Structural stability to improve activity has also been exploited for *Myceliophthora thermophile* laccase in [C_2_C_1_Im][EtSO_4_], where immobilization on agarose also prevented inactivation of this enzyme by 50% (Fernández-Fernández et al., 2014).

### Lysozyme

An experimental and MD simulation study by Ghosh et al. (2015) was able to show that the hydrodynamic radius of lysozyme decreased by more than a third when exposed to 1.5 M [C_3_C_1_Im][Br], while also conformational relaxation time decreases, implying structural stabilization and constraint by the ionic liquid. A preferential solvation of the protein surface by the cations over anions was demonstrated and further confirmed the much more compact structure and closer interaction between the two protein domains of lysozyme in ionic liquid, promoting the closed conformation (Ghosh et al., 2015). This finding underlines the importance of conformational entrapment of the protein by ions, with significant potential to alter reactivity.

Wijaya et al. (2016) highlighted the importance of the solvophobic effect and the role of ions by demonstrating favorable lysozyme stability and activity in highly concentrated or neat, non-aqueous solutions of protic ionic liquids. For those ionic liquids containing hydroxyl groups (ethanolammonium-based), a similarity to the effect of glycerol and its lower dielectric constant in creating additional stability was postulated. These hydroxyl moieties can result in stronger hydrogen bonds and screening of charged groups (Pérez and Griebenow, 2000), consistent with the results seen for ADH in ionic liquids. The effect of stabilization and destabilization on lysozyme followed the Hofmeister series (Kumar and Venkatesu, 2014), and the increased role of anions appeared to correlate with the positive surface charge of this enzyme (Wijaya et al., 2016). In a follow-up study Wijaya et al. (2018) further underlined the activity-increasing influence of reduced ion-protein interactions on lysozyme, as promoted by kosmotropic (ordering) ions, relative to the opposite impact that chaotropic (disordering) ions have on activity (Wijaya et al., 2018).

### Other Proteins

#### Cytochrome c

Complementing the finding of positive influence of hydroxyl groups on the structural stability of lysozyme, Papadopoulou et al. (2016) found hydroxyl ammonium based ionic liquids to mitigate the denaturing effect of H_2_O_2_ on the metalloprotein cytochrome c and increase the catalytic efficiency, relative to the buffer system. The combination of a more hydrophilic, and thus more chaotropic cation, such as hydroxyl alkyl ammonium cations, in combination with a kosmotropic anion was proposed to enhance cytochrome c peroxidase catalytic activity through perturbation structure and especially of the haem center. A considerable decrease in activation energy, and resultant increase in reaction rate, was observed when chaotropic cations were used (Papadopoulou et al., 2016).

Ikeda et al. (2018) used a bi-phasic system of [P_4, 4, 4, 4_][TMBS] (2,4,6-trimethylbenzenesulfonate) and buffer to show a favorable residence distribution of reduced and oxidized cytochrome c in the buffer and the hydrophobic ionic liquid phase, respectively. Using temperature gradients and applying a potential, cytochrome c could be reversibly transferred between phases. They found the oxidized form of cytochrome c, which resides in the IL, was more thermodynamically stable (Ikeda et al., 2018).

#### Tyrosinases

Tyrosinase catalyzes the oxidation of tyrosine through a copper active site and is connected to processes as diverse as melanin production and the over-ripening of fruits. In this regard, mainly kinetic mechanisms and the inhibition of the active site are of interest. Heitz and coworkers carried out a combined experimental kinetics and molecular docking study on mushroom tyrosinase in imidazolium-based cations (Heitz and Rupp, 2018; Heitz et al., 2018), with anions covering the range of the Hofmeister series (Kumar and Venkatesu, 2014). An increase in hydrophobicity of the ions led to an increase in preferential site interactions with the protein surface and active site. The anion [NO_3_] had the least impact on activity and was equally distributed on the enzyme surface. In contrast, the anion [MeSO_3_] had three preferential sites of interaction, which were dominated by positively charged and polar amino acids. The presence of fluorine atoms in [TFMS] had significant impact on the anion behavior in comparison to [MeSO_3_], and this fluorinated ion was mainly localized around the active site. The most detrimental ion to activity was [NTf_2_], which showed both competitive inhibition and preferentially interacted with active site residues, due to its hydrophobicity and electronegative atom composition. A similar preferential interaction within the active site was found for all cations, regardless of alkyl chain length, but an increasing hydrophobicity of the cation was correlated to a slight energy decrease in the docking values, indicating worse predicted binding (Heitz and Rupp, 2018; Heitz et al., 2018).

#### Transaminases

Transaminases are catalysts for the production of enantiomerically pure compounds, and in this regard they are similar in value to ADH for biotechnological applications. Again, poor solubility of the substrate or product in water is an issue, that is mainly overcome by the use of organic solvent. An alternative to overcoming the issue of inactivation in organic solvent by applying ionic liquids has been not by using them as a replacement for the organic phase, but to coat the enzyme in ionic liquids. Grabner et al. (2018) demonstrated an improved method for the coating of enzymes by ionic liquids for application in organic solvents by deploying Ω-transaminases. Co-lyophilisation of enzyme, ionic liquid ([AMIM][Cl] or [C_2_C_1_Im][Br]) and buffer yielded the best results, affording an 8-fold higher activity than compared to that of the free enzyme. The [C_2_C_1_Im][Br] coating was more favorable in increasing the yield for both enzymes in all solvents tested, relative to [AMIM][Cl], with the exception of the yields under the organic solvents Et_2_O and MTBE for one of the assayed enzymes (Grabner et al., 2018).

#### Selenate Reductase

Selenate reductase catalyzes the NADPH-dependant reduction of selenate and selenite to elemental selenium, which has multiple applications including in batteries, glass production, and solar cells. In a study by Mesbahi-Nowrouzi and Mollania (2018) two imidazolium-based ionic liquids were tested and the enzyme showed increased residual activity (~110%) in low concentrations of [C_2_C_1_Im][Br] [5–10% (v/v)] relative to buffer, which linearly decreased to 30% residual activity in 30% (v/v) ionic liquid. The thermal stability in this ionic liquid was also increased, indicating stabilization of the enzyme structure. For [C_4_C_1_Im][Cl] a linear decline in activity over all concentrations was observed (Mesbahi-Nowrouzi and Mollania, 2018), but this could be recovered on diluting the solution, suggesting an inhibition effect. Both ionic liquids also had an impact on the size of selenium nanoparticles that were produced by the enzyme, compared to the absence of ionic liquid.

#### Carbonic Anhydrases

Carbonic anhydrase enzymes have been attracting substantial interest because of their ability to “fix” CO_2_ through hydration. These enzymes thus have potential in carbon capture, provided they can be made robust enough to withstand the conditions needed, so thermotolerant and halotolerant variants have been attractive targets. To this aim, rational engineering of a mesohalophilic carbonic anhydrase has been achieved to generate extremely halotolerant variants, and subsequently the activity tested in the ionic liquids ethanolammonium formate (ETAF), ethyl ammonium formate (EAF), ethyl ammonium nitrate (EAN) and [C_1_C_1_Im][Me_2_PO_4_] (Warden et al., 2015). The ETAF protected against thermal unfolding for the most halotolerant variants with increasing concentration, consistent with the effects seen for lysozyme (Wijaya et al., 2016), and cytochrome c (Papadopoulou et al., 2016). The other ionic liquids tested had only a moderate impact on thermal stability of the extremely halotolerant enzyme.

## Future of Proteins and Enzymes in Ionic Liquids

The scope with which ionic liquids have already been utilized with proteins and enzymes is substantial with many exciting application areas already emerging. This includes the ability to analyse and sequence recalcitrant proteins (Plowman et al., 2014; Deb-Choudhury et al., 2016), the use of enzyme-ionic liquid solutions for the restoration of heritage materials (Hrdlickova Kuckova et al., 2014), the production of ionic gel-like materials (ionogels) that could be used in applications as diverse as biocatalysis, batteries, environmental applications, and soft robotics (Ribot et al., 2012; Shamsuri et al., 2012; Silva et al., 2012, 2013; Wang et al., 2013a; Kapoor and Kundu, 2016; Iqbal et al., 2017; Singh et al., 2017; Grollmisch et al., 2018; Yao et al., 2018), and extension to the spinning, casting, and 3D-printing of reprocessed materials (Phillips et al., 2005; Gupta et al., 2007; Meli et al., 2010; Deng et al., 2014; Gunasekera et al., 2016; Zavgorodnya et al., 2017; Gunasekera, 2019). Bio-sensing and stimuli-responsive applications also show significant scope (reviewed in Kavanagh et al., 2012). Clearly, there are still many unanswered questions and challenges in the selection and use of the correct ionic liquid for the correct application to ensure effective and productive outcomes.

### Challenges With Protein Processing

A number of challenges still need to be progressed in the reformation of protein-based materials, especially because of their applications in biotechnology and tissue engineering (Dal Pra et al., 2005; Park et al., 2006; Gupta et al., 2007; Rouse and Van Dyke, 2010; Vijayaraghavan et al., 2010; Silva et al., 2012, 2014; Kapoor and Kundu, 2016; Tran et al., 2016; Egorova et al., 2017; Iqbal et al., 2017, 2018a,b; Mahmood et al., 2017), with work being carried out to develop practical processing techniques. Among these are the issues with ionic liquid toxicity (Zhao et al., 2007; Ostadjoo et al., 2018), and by extension removal (Gupta et al., 2007; Silva et al., 2012, 2013; Iqbal et al., 2017), to ensure that the final materials are reliably biocompatible. Already the work on more biocompatible ionic liquids, such as those based on either cholinium cations or amino acid anions shows promising results, and is an area that should be further explored, not only in the context of biocompatibility, but also in sustainability. Primary human epidermal keratinocytes have been demonstrated as being able to grow on patterned films of silk, spin-coated from a 7.5% (w/w) solution of [C_4_C_1_Im][Cl] and 25% (w/w) water (added to reduce viscosity) (Gupta et al., 2007), and this study illustrated that tracking characteristic ionic liquid peaks in the ATR-FTIR spectrum (in this case, 1,463 cm^−1^ of [C_4_C_1_Im][Cl]) before and after methanol treatment, is able to confirm successful ionic liquid removal and simultaneous development of the β-sheet structures (indicated by a shift in the amide I peak from 1,650 to 1,622 cm^−1^).

Several studies have investigated the stability of both silk and keratin during their processing with ionic liquids and both proteins are quite often found to be degraded upon regeneration, either at the molecular level or with respect to secondary structural elements (Goujon et al., 2012; Idris et al., 2013; Ji et al., 2014; Cheng et al., 2015; Zheng et al., 2015; Liu et al., 2017; Zhang et al., 2017a,b). For keratins, decomposition of the disulfide linkages within the protein is expected as a natural part of dissolution, as without this the protein chains are too strongly bound. The high temperatures (>100°C) required for dissolution of keratin in ionic liquid provide an impetus for decomposition, and the temperature impact has been systematically studied by characterization of amino acid compositions of regenerated wool (Ghosh et al., 2014). Degradation of cysteine (comprising initially about 10% of the total keratin content) with some oxidative formation of cysteic acid and other derivatives increases with increasing temperature, with less than half remaining after 30 min treatment at 150°C (Ghosh et al., 2014). Cleavage of the disulfide bonds was attributed, at least in part, to the attack of chloride ion from the [C_4_C_1_Im][Cl] solvent. Variation both in the degree of retention of disulfides and presence of free sulfhydryl groups is seen with changing ionic liquid, with the dissolution capability mirroring the capacity to cleave the disulfide bonds and inversely proportional to the recovery percentage on regeneration (Zhang et al., 2017a). A minimum level of 65% cleavage was proposed as a benchmark requirement for adequate keratin dissolution, based on these data. A minimum temperature of 110°C for dissolution in [C_2_C_1_Im][Et_2_PO_4_] was also necessary.

The biggest issue with silk degradation seems to lie in the serecin removal stage, which is common to all silk processing methods. The prolonged heating of silk has an effect on the level of degradation observed, similar to keratin. To overcome this issue, Lozano-Pérez et al. (2015) utilized ultrasonication to dissolve silk in ionic liquids and determined the level of peptidic chain fragmentation with SDS-PAGE. Where conventional heating of silk in ionic liquid can take several hours for full dissolution, the use of ultrasound reduced this time substantially with [C_2_C_1_Im][Cl], reaching 23 ± 0.3% w/w in 17 min. SDS-PAGE analysis, tryptic degradation, and HPLC/MS confirmed that the silk treated with sonication in ionic liquid retained both much more of its integrity and, in addition, the lower molecular weight components of the silk, indicating a reduction in the thermal degradation of the silk. Combination of ultrasonication with other tailored and characterized dissolution methods (Wang et al., 2013b), may provide much improved methodology for the preservation of silk structure and properties going forward, along with enhanced recovery and reuse of the ionic liquids involved.

### Biomaterials and Composites Formation

The access to ionic liquid-soluble proteins, and other recalcitrant biomass, has naturally led to the production of composites in order to create materials with a great depth and breadth of physical and chemical properties (Mahmood et al., 2017). Popular have been combinations of protein and cellulosic derivatives (Xie et al., 2005; Hameed and Guo, 2009; Wu et al., 2009; Shang et al., 2011; Wang et al., 2013a, 2014; Yao et al., 2014a,b; Zhang et al., 2014; Kammiovirta et al., 2016; Tran and Mututuvari, 2016; Tran et al., 2016; Stanton et al., 2018), alongside other sugar polymers such as chitosan (Silva et al., 2012; Tran and Mututuvari, 2016), chitin (Park et al., 2006), and starch (Leroy et al., 2012). Both protein structuring and macrostructural morphology provide insight into the impact of ionic liquids in the formation of these materials.

Initial approaches to regeneration of keratin already focussed on composite formation with cellulose (Xie et al., 2005), with regeneration by soaking in methanol overnight. Fiber structuring was not seen for 1:5 mixtures of keratin (10 wt% in [C_4_C_1_Im][Cl]), with blending resulting instead in a homogenous membrane, as visualized by SEM. Cellulose acetate (CA) composites with wool at 20 and 40 wt% CA regenerated in water, in contrast, were less homogenous, although separate composites could not be easily distinguished (Hameed and Guo, 2009), neither at these or higher CA:wool ratios of 60 and 80 wt%, respectively. For these higher CA ratios, DSC analysis indicated a single phase, with significantly higher glass transition temperatures, along with increased thermal stability, relative to the pure regenerated components. This implies cooperative interactivity and new, strong hydrogen bonding between the wool and CA components, and changes to the hydrogen bonding were confirmed by FTIR. Industrial chicken feathers and cellulose (5 wt% in [C_2_C_1_Im][OAc]) in ratios from 70:30 to 0:100, respectively, were wet-spun and coagulated in ethanol (Kammiovirta et al., 2016). At 10:90 ratio, the strength and tenacity of the spun fiber was at its highest, and reinforced the fiber strength over pure cellulose. FTIR analysis suggested an unfolding of the β-sheets of the protein in the regenerated material. Characterization by SEM indicated a lack of phase separation, as per previous studies, however, keratin was unevenly distributed at the highest ratios and afforded a more porous structure, ascribed to asynchronous regeneration relative to cellulose. With this in mind, this result highlights how changes to the ionic liquid solvent may impact on regenerated properties, and how also tuning the solvent might be exploited.

Tran and coworkers have carried out the most extensive experiments to date on keratin-based composites formed from [C_4_C_1_Im][Cl], and regenerated with water with the ionic liquid recycled (Tran and Mututuvari, 2016; Tran et al., 2016). Similar to previous composite studies, the keratin-sugar-based composites had improved mechanical properties and were homogenous by SEM analysis, although displayed increased roughness with an increasing keratin content. Different sources of keratin, due to differences in their underlying primary and secondary structures, afforded different microcrystalline structures, as assessed by SEM, and their composites displayed different antimicrobial activities (Tran et al., 2016). This latter property was linked to the differences in secondary structure. On either chitosan or cellulose addition (Tran and Mututuvari, 2016; Tran et al., 2016), the α-helix content of the keratin reportedly dropped or stayed consistent, with an increase in β-sheet structuring, attributed to specific stabilization from the hydrogen-bonding of the sugar hydroxyl-moieties.

Silk-based biocomposites have attracted the strongest attention of the biocomposite studies, particularly in understanding the conditions for appropriate regeneration. Silk-cellulose composite films from [C_4_C_1_Im][Cl] were exposed to methanol vapor and then dried under vacuum (Zhou et al., 2013), in contrast to non-composite silk films that are commonly exposed to humid conditions as part of their processing method (Li et al., 2015). Subsequent SEM analysis indicated that a more fibrous microstructure with larger pores was observed with increasing silk loading. Cellulose content drove the formation of β-sheets and turns, as indicated by the change from 13 ± 4% in the pure silk sample to 41 ± 9% in the 75:25 cellulose-silk film, monitored by using deconvolution of FTIR spectra. The shift in the β-sheet peak from 1,629 to 1,623 cm^−1^, attributed to intramolecular and intermolecular β-sheets respectively (Hu et al., 2006), suggested that decreasing the silk content and increasing the cellulose content promotes intermolecular associations between neighboring fibroin polymer chains. Alternatively, this could be ascribed to the interaction of nearby cellulose chains with the hydrogen bonding network of the β-sheets (Zhang et al., 2016a). The eradication of XRD peaks associated with silk I (2θ = 8°) and silk II (2θ = 28°) structures suggest a reduction in silk ordering, however previous WAXS data for silk have shown a peak at 2θ = 21° that could be masked by the cellulose peaks (2θ = 22.4°) (Stanton et al., 2018), especially considering the continued presence of a strong β-sheet peak in the FTIR (Phillips et al., 2004).

The mechanical strength of these composite films was also correlated with cellulose content with the dry films ranging from 49.8 ± 7.4 MPa breaking stress when the silk-cellulose ratio was 75:25 to 146.9 ± 18.8 MPa for pure cellulose. The hydrated films were much weaker ranging from 0.9 ± 0.1 MPa to 10.7 ± 3.1 MPa, respectively. A qualitative inspection of cell viability using murine fibroblast cells L929 was carried out for the composite films, with cell density correlated with silk content. Further work in this area would help to establish key composite parameters for viability of cell adhesion and proliferation.

The effects of different ILs on composite films made from a 9% w/w cellulose and 1% w/w silk solution (Table 3), indicated that [C_4_C_1_Im][Br] and [C_4_C_1_Im][MeSO_3_]-derived composites formed translucent and brittle films, while all the others were solid and clear (Stanton et al., 2018). The brittle films exhibited the highest β-sheet content (58.6% for [C_4_C_1_Im][Br] and 58.9% for [C_4_C_1_Im][MeSO_3_], respectively), as determined by Fourier self-deconvolution of the amide I region of the FTIR spectra of the films and the H-bonding capability of the anion was inversely proportional to the degree of β-sheet formation. The rationale provided was that the high β-sheet formation was driven by stronger interactions between the biopolymer components relative to those with the anions of the ionic liquid, allowing the reformation of original, albeit disrupted, β-sheets, as well as new ones upon coagulation.

Surface topography of silk-cellulose blends seems also to be very closely linked to anion character; regeneration with chloride-ionic liquid displayed a smooth surface for the biocomposite film, whereas the acetate-derived film had small pores and the [MeSO_3_] and bromide-derived films both showed a fibrous structure. A role for the cation was indicated by X-ray scattering data, where moving from [AMIM][Cl]to [C_2_C_1_Im][Cl] increased the amorphous nature of the blend, shown by the backbone spacing between the cellulose and the silk. X-ray scattering peaks for [C_2_C_1_Im][OAc] broadened, indicating an increase in the interaction with the anion and a resultant rise in the blending of the silk and cellulose chains in the film. For the [C_4_C_1_Im][MeSO_3_] and the [C_4_C_1_Im][Br] there were clear peaks for both the cellulose crystallites and the β-sheets, which, combined with the high crystallinity shown in the FTIR spectra, are indicative of separated microphases. This could be the basis for the brittle nature of the films made with these ionic liquids. Previous studies with [C_4_C_1_Im][Br] have demonstrated that it is a poor solvent for silk providing a rationale for microphase formation (Phillips et al., 2004; Mantz et al., 2007;Stanton et al., 2018).

The similar solubilising properties [C_4_C_1_Im][OAc] possesses for chitin and silk also allows them to be blended into a single structure (Park et al., 2006). This builds on the previously determined solubility of chitosan in ionic liquids (initially by Mantz et al., 2007) toward the production of a blended hydrogel for evaluating the seeding of human Dermal Fibroblasts (hDFs). The different ratios of chitin and silk (Table 3) have differing properties; primarily a greater β-sheet structure when the silk to chitosan ratio was 70:30, as determined by FTIR measurements of the amide peaks. The higher silk ratio imbued the hydrogel with greater rigidity and superior elastic behavior over the frequencies tested. The chitosan in these hydrogels exhibited changes to its secondary structure that, along with the homogenous structure observed in SEM, indicate physical interactions between the two biomaterials most likely in the form of either direct hydrogen bonding or ionic liquid anions being locked into the structure of the hydrogel.

### Ionogels and Ionic Liquid-Mediated Entrapment

Ionogels or gel-polymer electrolytes (GPEs) are conducting gel-like materials that can arise from confinement of ionic liquids within ordered matricies (Marr and Marr, 2016). Such gels have found potential application in the areas of environmental cleanup (Billeci et al., 2018), and batteries (Cerclier et al., 2015), with further scope for areas where flexible conductive gels would be useful, such as soft robotics and smart bioelectrochemical devices (Fujita et al., 2012). Desirable properties of such gels include responsiveness to stimuli [e.g., thermoresponsive (Ribot et al., 2010) and electrochromic gels (Benedetti et al., 2015)] and self-healing abilities. Proteins and their composites offer nanostructuring opportunities for ionic liquid ions through their hydrogen-bonding network and ionic interactions, consistent with the reported gel-formation of some wool and silk materials prior to regeneration.

Ion jelly®, formed from gelatine and ionic liquid (including a range of cholinium-based ionic liquids), has been shown to be extremely versatile, with applications including selective membranes, gas separation, conductive coatings for textiles, development of antimicrobial fibers, solid-state electrochromic systems, and as a gas sensor (Vidinha et al., 2008; Nuno et al., 2011; Couto et al., 2013, 2015; Rana et al., 2013; Santos et al., 2013; Carvalho et al., 2014; Benedetti et al., 2015). Gelatine-based ionogels have also been partnered with silver oxide nanoparticles to generate microbe-resistant and highly stretchable materials that are also self-healing and have shape-memory (Singh et al., 2017). Gelatine with a propionic acid-functionalised ionic liquid [(C_2_CO_2_H)C_1_Im][Br] and embedded Eu^3+^ ions retained luminescence, in contrast to aqueous solutions of Eu^3+^, and could be utilized in UV-emitting LEDs (Li et al., 2014). Entrapment of enzymes within ionic liquid gels also offers an alternative to standard immobilization, with the benefits imparted by the ionic liquid. The protection of horse-radish peroxidase by entrapment in Ion Jelly® formed from [C_2_C_1_Im][EtSO_4_] is one such example (Lourenço et al., 2011). Similarly, glucose oxidase was able to be stabilized and utilized as a gel-coating for the sensing of glucose with this system.

Hydrogel formation in silks is well established (Kapoor and Kundu, 2016), and has been extended to ionogel formation (Zhang et al., 2016a; Yao et al., 2018). By varying silk, water, and [C_2_C_1_Im][OAc] ratios, differing mechanical properties could be obtained with the best being 10% silk, 50% [C_2_C_1_Im][OAc] and 40% water (all w/w) (Yao et al., 2018). The major drawback to silk-IL-water hydrogels is the gelation times, which has also been an issue in previous work (Zhang et al., 2016a). The gelation times for the silk:[C_2_C_1_Im][OAc] (50% w/w): water (40% w/w) hydrogel ranged from 30 days for mixtures containing 5% w/w silk, to 9 days for 10% w/w silk. Depending upon amount of ethanol added to a gel with 10% w/w silk, the gelation times could be improved from approximately 40 h for 10% v/v in water to just over an hour with 60% v/v, with little difference in the mechanical performance of the final gel in the range of 0–40% v/v, with the exception of a slight improvement to tensile properties (except stiffness). Increasing ethanol concentration also increases the rate at which the β-sheet folding transition occurs, giving another mechanism for tuning the mechanical properties of silk hydrogels formed in this manner.

Beyond gelatine and silk, scope for developing ionic liquid-based ionogel formation with other proteins also remains (Zhu et al., 2016). Enzyme immobilization in the context of biosensors and stimuli-responsive materials, including entrapment in both ionogels and polymerisable ionic liquid matricies (Grollmisch et al., 2018), has been well-reviewed recently (Kavanagh et al., 2012; Zuliani et al., 2014; Marr and Marr, 2016; Zhang et al., 2016b). Commonly, sensors have utilized glucose oxidase (GOx) with a view to producing accessible glucose sensors, primarily in combination with imidazolium-based ionic liquids (Sharma et al., 2015). Lactate oxidase in [C_2_C_1_Im][EtSO_4_] has been similarly employed for lactate sensing (Khodagholy et al., 2012). Cellulases (Hosseini et al., 2018) and lipases (Suo et al., 2019) have recently been used as immobilized catalytic components, where additional functionalities such as magnetism can also be incorporated. As per other ionic liquid-protein applications, more biocompatible ionic liquids, such as those based on cholinium cations and amino acid anions, have been recently demonstrated as being useful in developing laccase-based biosensors (Zappi et al., 2018) and more generic enzyme-based sensors (Zappi et al., 2017). Certainly, as dissolution and gelation technology advances, tailored ionic liquid-based immobilization incorporating alternative biopolymers, and with a broader range of sensing capabilities, offers exciting and sustainable opportunities.

### Potential for Driving Reactions of Proteins

One of the future application areas for proteins will not only be the reprocessing of existing biopolymers, but the ability to derivatise these materials to create new materials properties. For Zein, proof of principle has been achieved in benzoylation (Biswas et al., 2006), and for silks sulfation has been effective (Liu et al., 2015). Such reactions could therefore readily be extended to other proteins. One key consideration, however, is how the ionic liquid solvent is organized around the protein (Hayes et al., 2015; Sprenger et al., 2017). Beyond the demonstrated impact of these interactions in creating the initial solubilisation, as noted through the extensive experimental work in this review, it has been well established that the organization of an ionic liquid around a potential reactive center plays a significant role in directing reactions at that center (Yau et al., 2008, 2009a,b, 2012, 2013; Tanner et al., 2013a,b; Keaveney et al., 2016, 2017, 2018; Hawker and Harper, 2018; Hawker et al., 2018; Schaffarczyk Mchale et al., 2018; Gilbert et al., 2019). The surface site interactions, leading to potential inner site interactions if hydrogen bonds are disrupted, are governed by the relative affinity of the ionic liquid ions for specific amino acids (Sprenger et al., 2017). Ionic liquids should thus be able to be tailored to enhance specific interactions, and, in addition, cooperative effects based on sequence are likely to generate differential organization, leading to different accessibility of reactive residues. In this way, specificity for particular sites on an enzyme could be engendered that can afford some selectivity in reaction. Further, where competitive reaction mechanisms exist, then another level of selectivity can be applied through the appropriate selection of ionic liquid (Tanner et al., 2013b; Yau et al., 2013; Hawker and Harper, 2018). Still, however, much further work needs to be done to develop the predictive rules for these interactions, although initial work has begun in this regard (Sprenger et al., 2017).

## Synopsis

The areas of application at the intersection of protein biochemistry, enzymology and ionic liquids are extensive and show promise for future applications. A variety of ionic liquids have been trialed with both structural proteins and enzymes, often with the exact set differing depending on purpose. The focus to date in all areas has been primarily on imidazolium-based ionic liquids, presumably due to their wide accessibility and established effectiveness. For most purposes involving solubilisation of a protein component, short chain substituents on the imidazolium have been most successful. Some other classes of ionic liquid cation are represented, including ammonium-based ionic liquids, pyrrolidiniums, and increasingly more functional ionic liquids such as protic ionic liquids, and/or bio-derived ionic liquids (including cholinium and amino-acid based species). The limited, but promising, studies utilizing the latter classes leaves a broad scope for future studies with good potential for even greener and more sustainable methodologies and applications.

The properties of regenerated biopolymers show variation with the ionic liquid used for dissolution, and the antisolvent used to generate the material. Films, membranes, gels and nanoparticles can all be formed under different conditions, although in pure biopolymers the secondary structure disruption on dissolution and imperfect reconstitution on regeneration, for example the increase in β-sheet formation in keratins and silks, can impact on properties. This can be mitigated to some extent by blending with sugar-based biopolymers, which are able to stabilize the protein structures and impart enhanced mechanical properties on the final composite, relative to the initial pure components, once regenerated.

Progress is also being made on the predictive understanding of ionic liquid-protein interactions in order to optimize them. In the enzyme sphere, the Hofmeister series provides an initial benchmark, with more detailed molecular simulation studies supporting a more detailed insight per enzyme feeding into more generalized principles. For solubility, increasingly sophisticated methods based on computational screening, such as use of COSMO to calculate ln γ values, appears to be effective, and other more general computational approaches are being developed that should allow more detailed interrogation of how structure is impacted by interactions with ions. A near future can be envisaged, whereby a pick-and-mix approach, included tailored binary and ternary mixtures, could be achieved to rapidly match a desired application.

## Author Contributions

All authors listed have made a substantial, direct and intellectual contribution to the work, and approved it for publication.

### Conflict of Interest Statement

The authors declare that the research was conducted in the absence of any commercial or financial relationships that could be construed as a potential conflict of interest.
